# The blood–brain barriers: novel nanocarriers for central nervous system diseases

**DOI:** 10.1186/s12951-025-03247-8

**Published:** 2025-02-26

**Authors:** Jiajun Liu, Ting Wang, Jian Dong, Yuan Lu

**Affiliations:** 1 State Key Laboratory of Green Biomanufacturing, Department of Chemical Engineering, Tsinghua University, Beijing, 100084 China; 2 Key Laboratory of Industrial Biocatalysis, Ministry of Education, Department of Chemical Engineering, Tsinghua University, Beijing, 100084 China; 3 Tianjin Industrial Microbiology Key Laboratory, College of Biotechnology, Tianjin University of Science and Technology, Tianjin, 300457 China

**Keywords:** Central nervous system, Blood–brain barrier, Brain-based drug delivery, Nanoparticles

## Abstract

**Abstract:**

The central nervous system (CNS) diseases are major contributors to death and disability worldwide. However, the blood–brain barrier (BBB) often prevents drugs intended for CNS diseases from effectively crossing into the brain parenchyma to deliver their therapeutic effects. The blood–brain barrier is a semi-permeable barrier with high selectivity. The BBB primarily manages the transport of substances between the blood and the CNS. To enhance drug delivery for CNS disease treatment, various brain-based drug delivery strategies overcoming the BBB have been developed. Among them, nanoparticles (NPs) have been emphasized due to their multiple excellent properties. This review starts with an overview of the BBB’s anatomical structure and physiological roles, and then explores the mechanisms, both endogenous and exogenous, that facilitate the NP passage across the BBB. The text also delves into how nanoparticles' shape, charge, size, and surface ligands affect their ability to cross the BBB and offers an overview of different nanoparticle classifications. This review concludes with an examination of the current challenges in utilizing nanomaterials for brain drug delivery and discusses corresponding directions for solutions. This review aims to propose innovative diagnostic and therapeutic approaches for CNS diseases and enhance drug design for more effective delivery across the BBB.

**Graphical abstract:**

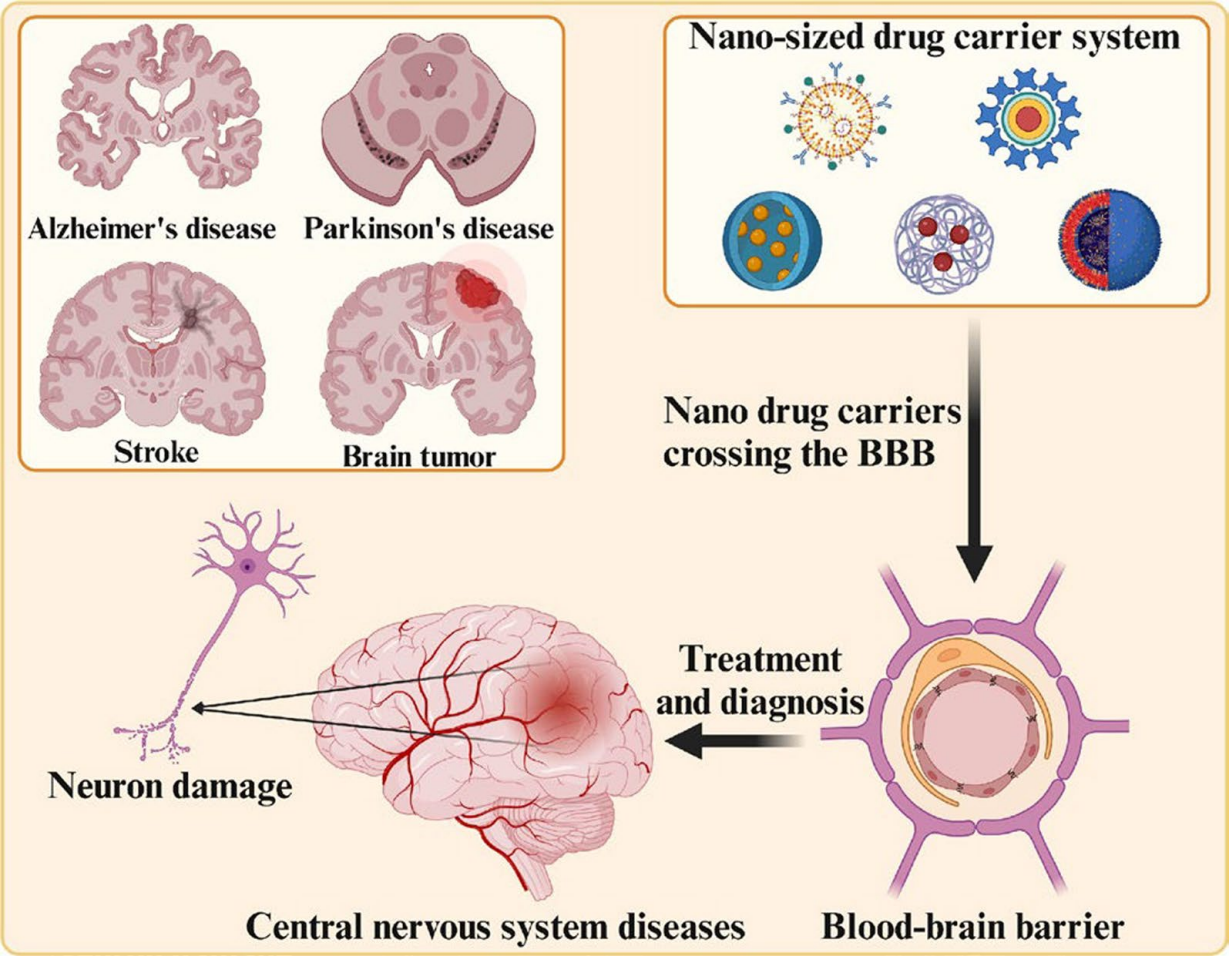

## Introduction

Diseases of the central nervous system (CNS), including Alzheimer's disease, Parkinson's disease, stroke, and brain tumors, have affected the normal lives of an increasing number of people worldwide in recent years[1–3]. However, most therapeutic drugs are unable to enter the brain parenchyma to exert their effects due to the presence of the blood–brain barrier (BBB), so more effective treatments need to be developed for central nervous system diseases. Therefore, an in-depth understanding of the physicochemical properties of BBB and its pathological changes in different CNS diseases is essential. The BBB is a highly selective semi-permeable barrier that exists between the CNS and blood. The primary role of the BBB is to control the flow of substances between the blood and the central nervous system according to the physiological needs of the brain. It safeguards the CNS from harmful toxins, pathogens, and foreign substances in the blood, while simultaneously supplying vital nutrients to brain tissue and eliminating waste products[4].

**Fig. 1:**
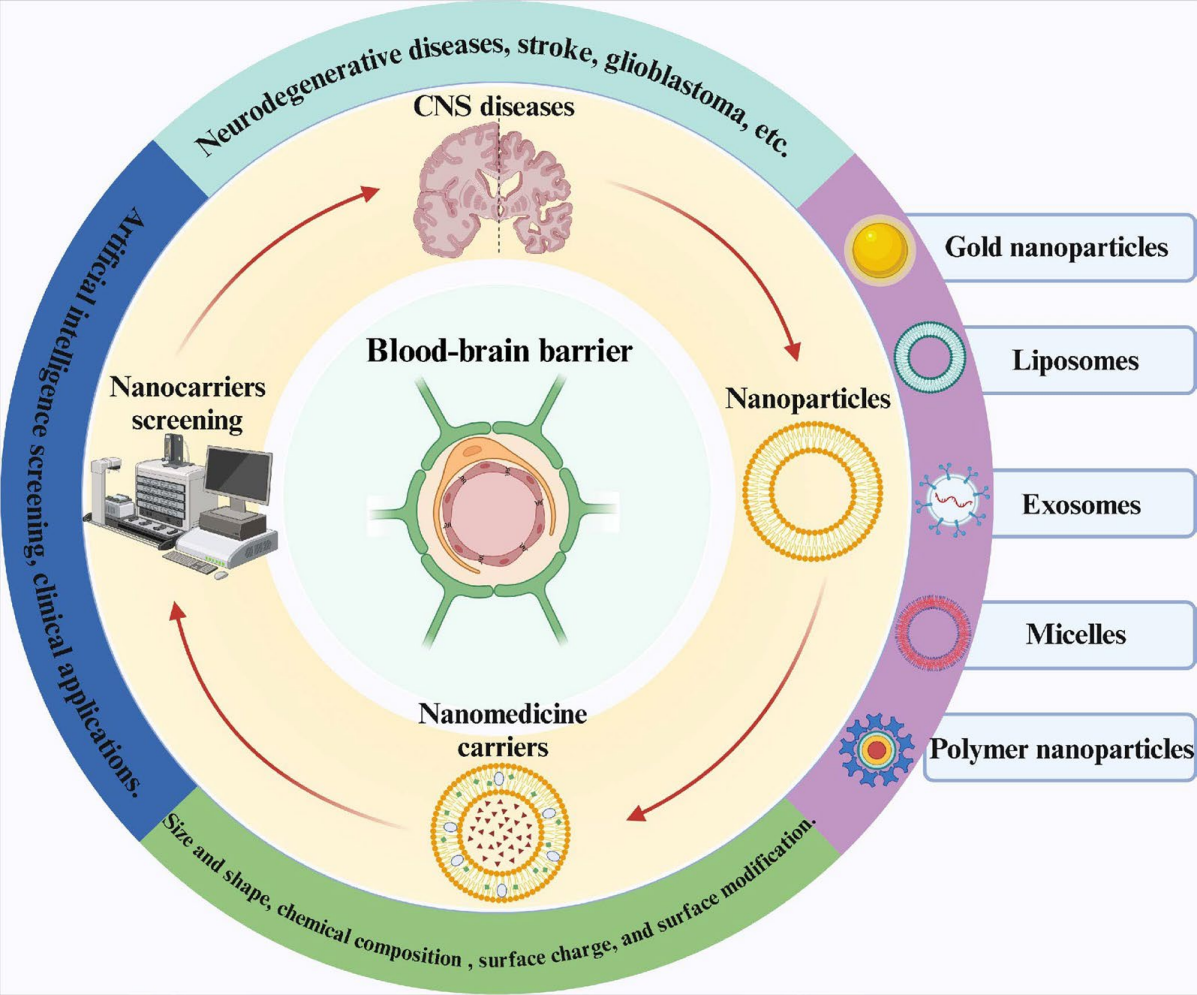
Design and screening of nanoparticles for drug delivery across the blood–brain barrier for treating CNS diseases. Created in BioRender

With the development of nanotechnology, there has been a breakthrough in the harmless passage of drugs through the BBB for the treatment of CNS diseases. Nanoparticles provide targeted therapeutic and diagnostic effects due to higher drug loading and bioavailability, lower dosing frequency, good biocompatibility and biodegradability, greater stability, fewer side effects, and less invasiveness[5, 6]. Encapsulation of drugs in NPs or coupling on their surfaces can be prepared as nanodrug delivery carriers[7]. Importantly, there are also nanoparticles that are not coupled to any drug and can be used as therapeutic agents themselves for specific diseases[8, 9]. Nanomaterials such as inorganic nanoparticles and polymeric nanoparticles are being designed and developed as safe, effective and practical drug delivery vehicles for crossing the BBB for the treatment and diagnosis of CNS diseases (Fig. 1). However, the use of nanoparticles for the treatment of CNS diseases still has several issues that are yet to be resolved. For example, inorganic nanoparticles, including gold and iron nanoparticles, are not easily degraded and can be neurotoxic when they accumulate in the brain[10, 11]. There is still a lack of preclinical in vitro blood–brain barrier models that can perfectly mimic the brain microenvironment, leading to limitations in the effectiveness of in vitro screening and practical application of nanomedicine carriers. Moreover, the production cost of nanocarriers is high, and the process is complicated, which makes it difficult to produce them on a large scale and apply them widely. To address these remaining issues, we also need to develop greener and more efficient ways to produce nanoparticles with higher biocompatibility. And to design an in vitro blood–brain barrier model more like the brain microenvironment to perform rapid and effective screening of these nanomedicine carriers with potential for clinical applications.

Against this background, this review provides an overview of the structure and function of the blood–brain barrier and discusses the abnormal changes in the BBB in different CNS diseases. It mainly summarizes the factors affecting the permeability of nanoparticles across the BBB and discusses them in depth. Unlike other reviews, this review provides a very complete summary of the endogenous and exogenous transport mechanisms involved in this process in the context of recent studies. The review also provides a specific categorization of nanoparticles used for the treatment of CNS diseases and discusses their respective strengths and weaknesses, as well as the latest applications in this field. Finally, unresolved issues in the field are summarized, and directions for addressing them are proposed accordingly.

## The BBB structure and function

### The discovery of BBB

**Fig. 2:**
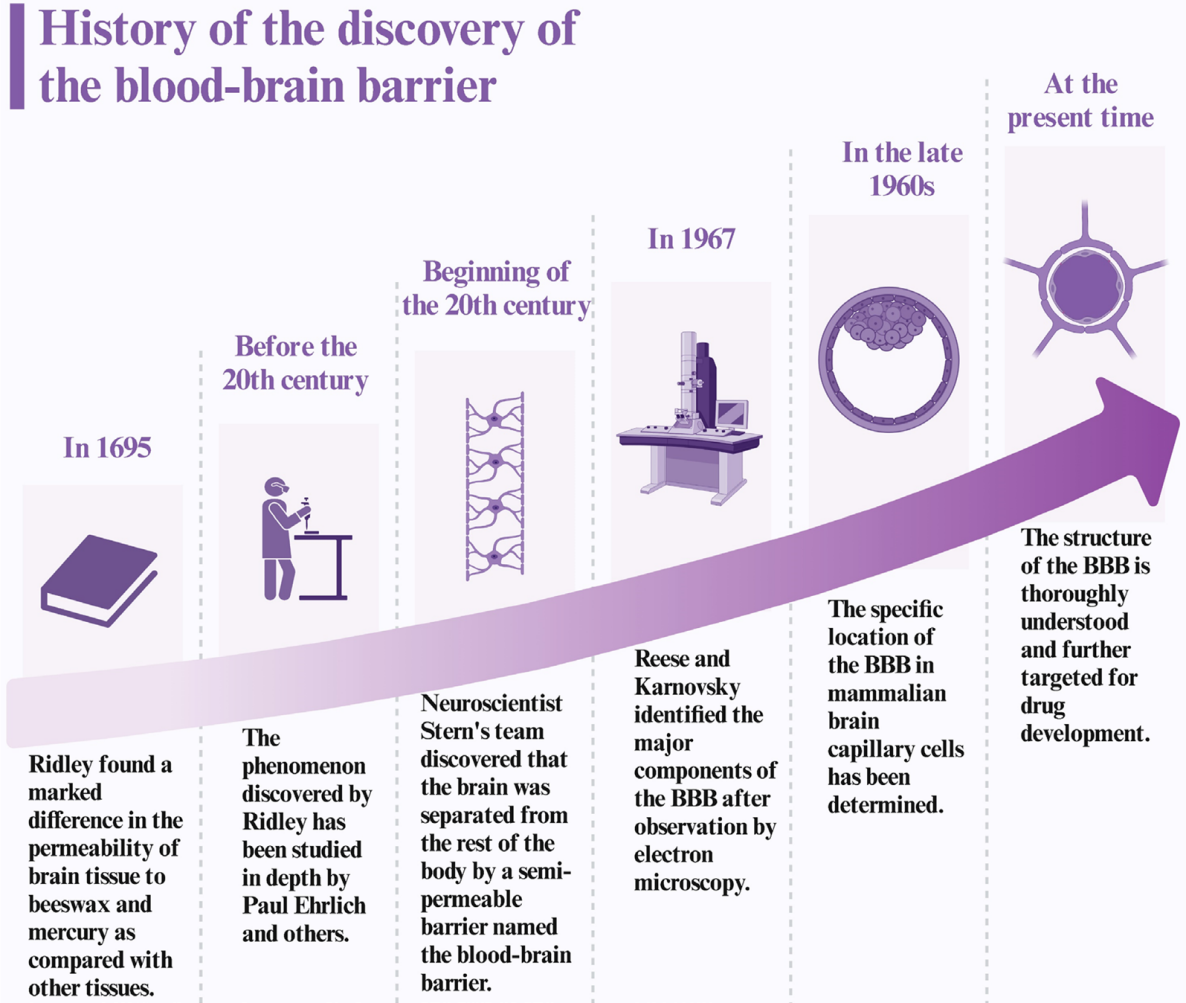
The blood–brain barrier's journey of discovery from 1695 to the present. Created in BioRender

The discovery of the blood–brain barrier has been a long process (Fig. 2). In his 1695 publication "Anatomy of the Brain," Ridley noted the distinct permeability differences of beeswax and mercury in brain tissue compared to other tissues, discussing this observation. Following this initial observation, pioneering researchers such as Paul Ehrlich further explored the permeability of different substances across the barrier separating blood from brain tissue. In the early twentieth century, neurologist Stern and his team, investigating substance transportation between the brain, cerebrospinal fluid, and peripheral blood, identified a semipermeable barrier separating the brain from the rest of the body, facilitating bidirectional movement, and named it the blood–brain barrier. and named it the blood–brain barrier[12]. In 1967, Reese and Karnovsky, through electron microscopy observations, determined that the BBB is primarily composed of astrocyte end feet and capillary endothelial cells[13]. In the late 1960s, researchers were able to pinpoint the specific location of the mammalian BBB within brain capillary cells[13]. It is now firmly established that the endothelial cells comprising the BBB create a selectively permeable barrier, facilitating the passage of essential nutrients from the bloodstream to the brain while effectively blocking the entry of potentially harmful substances into the brain parenchyma[14].

### Physiological structure of the BBB

**Fig. 3:**
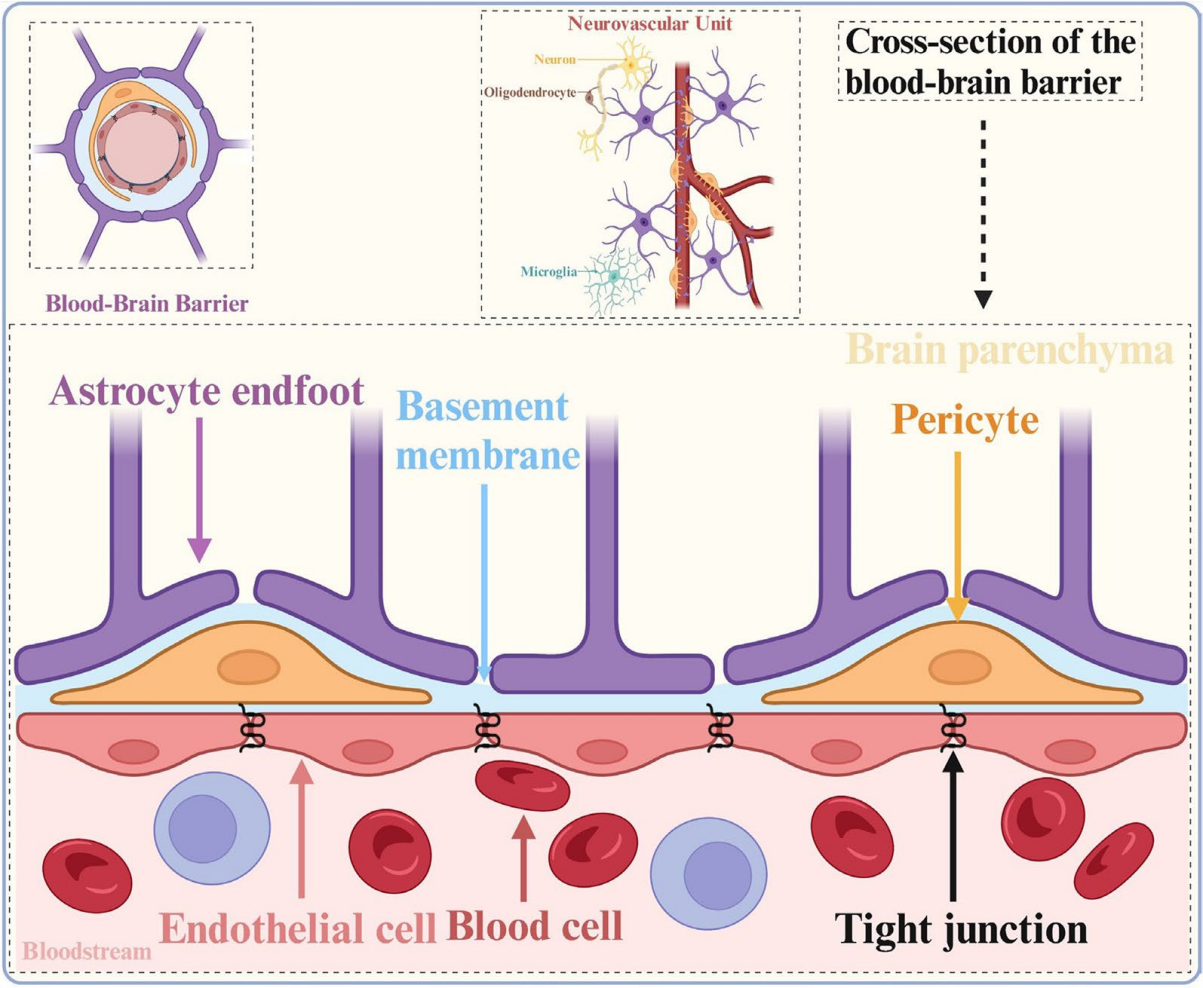
Structural diagrams of the blood–brain barrier and neurons. Created in BioRender

Several physiological barriers surround the CNS, including the BBB, blood-spinal cord barrier, multidrug-resistant proteins, blood-cerebrospinal fluid barrier, and arachnoid barrier (Fig. 3). These physiological barriers often prevent most drugs with clinical potential from reaching their target area in adequate concentrations [15]. A key factor in this process is the blood–brain barrier, which primarily comprises endothelial cells, astrocytes, and pericytes. Other involved elements include neurons, basement membranes, and microglia. These components, collectively known as the neurovascular unit (NVU), work together to support the normal functioning of the central nervous system.

#### Endothelial cells

Endothelial cells are a core component of the BBB, but endothelial cells in the BBB do not have the same structure and function as endothelial cells in other parts of the body[16]. In the BBB, endothelial cells are connected to each other by tight junctions and adherens junctions. Tight junctions, a collection of cytosolic and transmembrane proteins connected to the cytoskeleton, are key determinants of paracellular permeability[17]. Adherens junctions are mainly composed of cadherins and nectins, which act mainly on cellular integrity[18]. Adherens junctions bind to tight junctions and form distinct luminal and abluminal regions in endothelial cells, which can interact with each other through transcytosis. Endothelial cells in the BBB also express a variety of efflux transporter proteins that drain various lipophilic molecules from the brain back into the bloodstream[19].

#### Astrocytes

Astrocytes constitute the predominant cell type in the central nervous system of vertebrates. They are star-shaped, abundant, and multifunctional, guiding the migration of developing neurons. They have multiple appendages covering almost the entire brain capillaries surface. The endothelium of astrocytes wraps around blood vessels and forms a signaling pathway through various binding proteins attached to the basal lamina surrounding the vessels. Through this pathway, neuronal signaling can be delivered to endothelial cells, which is essential for the functional integrity of the BBB. Studies have demonstrated that properly regulating astrocyte function can enhance BBB performance and help repair its disruption following brain injury[20]. Astrocytes develop during late pregnancy from typical brain precursor cells and radial glia. This indicates that the initial processes of BBB formation are not regulated by astrocytes[21].

#### Pericytes

Pericytes are centrally positioned between neurons, astrocytes, and endothelial cells[22]. The proper development, stability, growth, and maintenance of the BBB hinge on the intricate interactions between pericytes and endothelial cells[23]. Pericytes contribute to regulating BBB permeability and cerebral blood flow, and they also participate in the biological clearance of harmful foreign compounds[24, 25]. As the pericyte surrounds endothelial cells and astrocytes, it often exchanges metabolites, ions, and messenger molecules among them. For example, knocking out PDGF-Rβ resulted in loss of blood–brain barrier function in pericyte-depleted mice, demonstrating the importance of pericytes in maintaining the functional integrity of the BBB[26]. Thus, pericytes are crucial to the physiology of diseases associated with the BBB.

#### Basement membrane

The basement membrane (BM) also plays an important role in regulating the BBB permeability. The BM connects cells, supports intercellular communication, and interacts with the extracellular matrix to control the BBB permeability[27]. Within blood vessels, the BM plays a pivotal role as an intermediary for signaling and functions as a barrier, effectively preventing the entry of chemicals and cells into brain tissue. The basement membrane degradation by matrix metalloproteinases plays a crucial role in causing BBB damage and promoting leukocyte leakage in a variety of CNS diseases.

#### Microglia

Microglia are specialized neuroglial cells primarily distributed throughout the spinal cord and brain. In brain tissue, microglia constitute approximately 5–20% of the total number of glial cells [28]. Microglia functions by enhancing the body's immune response, phagocytosing foreign particles, repairing damaged brain tissue, and transmitting intercellular signals. Additionally, research has shown that microglia can regulate tight junction expression, thereby enhancing the integrity of the BBB [29]. Hence, the properties of the BBB are sustained by the collaborative interactions among the constituents of the neurovascular unit.

### The BBB mechanisms and functions

The main physiological roles of the BBB include providing nutrients to the brain and maintaining ionic balance, shielding the brain from external neurotoxins, and regulating neurotransmitter levels. It serves as a crucial regulatory mechanism within the brain. The BBB primarily prevents certain macromolecules and toxic substances from entering the brain by altering the permeability of cerebral capillaries. Moreover, it restricts drugs' entry and therapeutic effectiveness for treating CNS diseases. The BBB regulates the movement of water and salts from the blood to the extracellular fluid, contributing to the brain's stable overall volume[30]. In other body tissues, extracellular fluid is generated through capillary leakage. If the BBB is compromised by damage or infection, water and salts can infiltrate brain tissue, causing an increase in intracranial pressure and swelling. Thus, the BBB protects the brain from fluids entering from other parts of the body that can cause health problems.

**Fig. 4:**
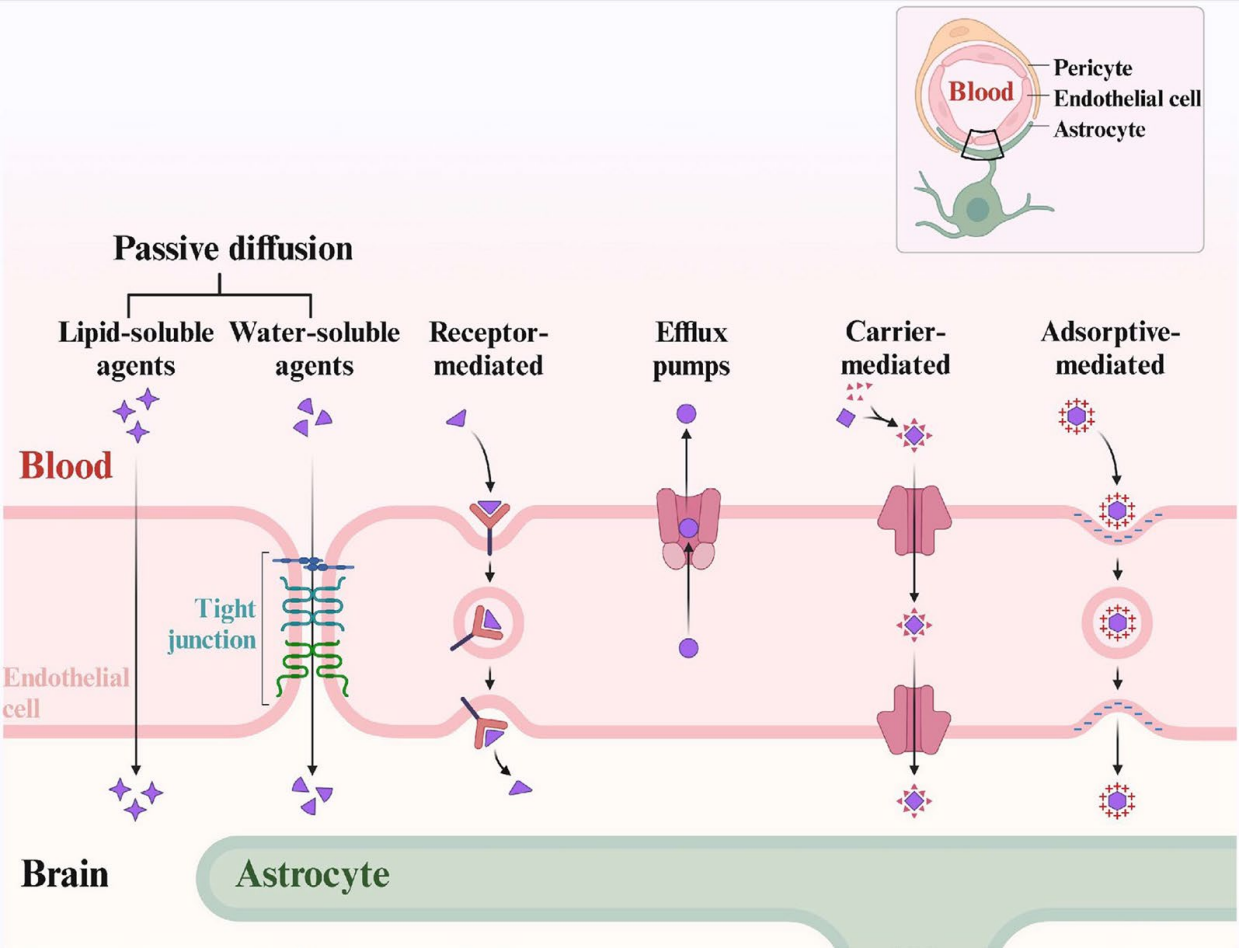
Five pathways for substances to enter the blood–brain barrier. The main ones include passive diffusion, carrier-mediated transcytosis, adsorptive-mediated transcytosis, receptor-mediated transcytosis, and efflux pump[31, 32]. Created in BioRender

The brain's need for nutrients and energy is high, even though the transit of substances through the BBB is greatly restricted. Hence, the brain can absorb endogenous substances via diverse passive or active transport routes. In general, under physiological conditions, substances can cross the BBB through five pathways, including passive diffusion, carrier-mediated transport, adsorptive-mediated transcytosis, adsorptive-mediated transcytosis, and efflux pumps[31, 32] (Fig. 4).

#### Passive diffusion

Passive diffusion is a nonspecific, energy-free mode of transport that moves small molecules down their concentration gradient. It mainly includes lipid-soluble agents and water-soluble agents. Small molecules of water-soluble substances can cross the BBB through paracellular passive diffusion, moving down an inverse concentration gradient across tight junctions. Lipophilic, nonpolar, and low molecular weight substances such as oxygen, carbon dioxide, and alcohol can pass through the cardiovascular cell membrane and enter the BBB through a cross-cellular pathway[33].

#### Carrier-mediated transcytosis

Carrier-mediated transport can be divided into facilitated transportation and secondary active transport. Nutrients like glucose, amino acids, and nucleotides can be transported via this mechanism[34]. In this transportation route, the substance initially attaches to a particular transporter located on the lateral aspect of the canal lumen. Following a conformational alteration, it subsequently gains entry into the brain parenchyma.

#### Adsorptive-mediated transcytosis

The endothelial cell membrane of the BBB is coated with a glycocalyx composed of heparan sulfate proteoglycans, which bear numerous negative charges. The presence of sialo glycoproteins and sialo glycolipids also gives the BBB surface a negative charge. Therefore, the attraction between positively charged cationic molecules and the negatively charged surface of the membrane can assist in their transfer into the brain parenchyma[35].

#### Receptor-mediated transcytosis

Receptor-mediated transcytosis (RMT) begins when the ligand attaches to particular receptors on the luminal side of vascular endothelial cells, triggering membrane invagination followed by endocytosis. Since receptors for elements such as iron, insulin, and leptin are highly expressed in the lumen, these elements can be transported via RMT [36].

#### Efflux pumps

Efflux pumps, proteins integrated into the endothelial membrane, harness the energy derived from ATP hydrolysis to facilitate the transport of substances across the cell membrane. Thus, they allow substances to outflow against the concentration gradient, expelling unwanted substances. The key efflux pumps present in the BBB comprise P-glycoprotein (P-gp), the breast cancer resistance protein, and various drug resistance-associated proteins[37].

## Structural abnormalities of the BBB in CNS disease

When multiple CNS diseases (such as Alzheimer's disease, multiple sclerosis, and stroke) occur, the resulting pathological responses (including inflammation, lipid peroxidation, and excitotoxicity) destabilize the BBB, leading to its dysfunction[38, 39]. Understanding the structural abnormalities and dysfunctions of the BBB in CNS diseases can provide a strong basis for diagnosing, monitoring, and treating these conditions.

### The BBB in neurodegenerative diseases

#### The BBB in Alzheimer's disease

**Fig. 5:**
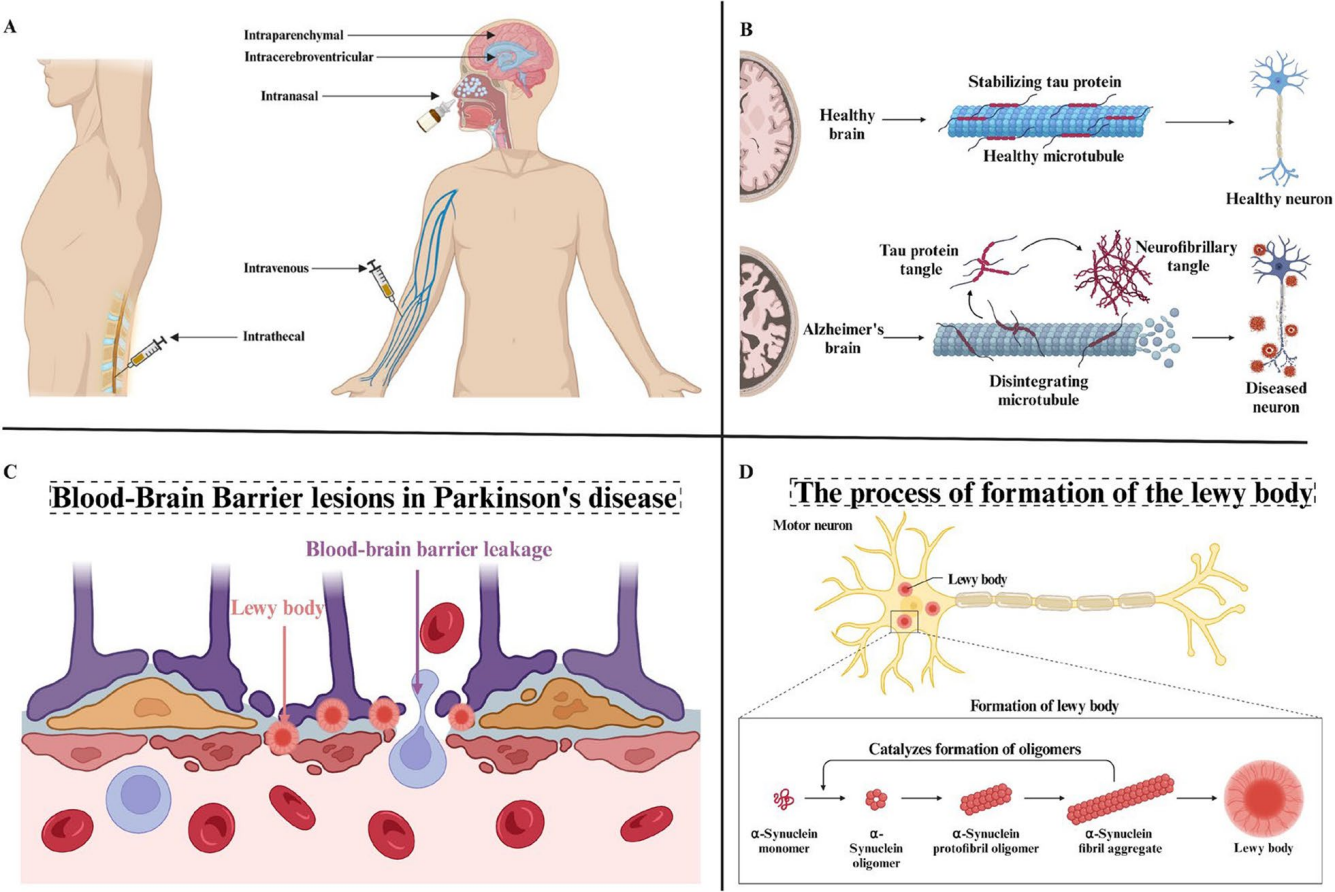
**A** Different routes of administration of nanotherapeutics to the CNS for the treatment of Alzheimer’s disease. Reprinted permission from Ref.[44]. Copyright 2024, Muolokwu, Chaulagain, Gothwal, Mahanta, Tagoe, Lamsal and Singh. **B** Comparison of healthy and diseased microtubule, brain and neurons in Alzheimer's disease. Created in BioRender. **C** Blood–brain barrier leakage after the onset of Parkinson's disease. Created in BioRender. **D** Motor neuron changes and lewy body formation in the pathogenesis of Parkinson's disease. Created in BioRender

Primary pathological features of Alzheimer's disease include extracellular senile plaques composed of amyloid beta (Aβ) fibers and intracellular neurofibrillary tangles formed by tau proteins[40]. Misfolded amyloid proteins compromise the BBB integrity, impairing its ability to perform its normal functions[41, 42]. The two-hit vascular hypothesis of Alzheimer's disease proposes that BBB dysfunction allows neurotoxins to enter the brain parenchyma, which in turn leads to neuroinflammation[43]. Changes in the BBB associated with Alzheimer's disease primarily include endothelial abnormalities such as reactive gliosis, mitochondrial damage, alterations in the extracellular matrix, disrupted tight junctions, and both molecular and functional changes in astrocytes. Nanoparticles encapsulating drugs can be delivered to the CNS for treating Alzheimer's disease through various pathways, including intravenous, intranasal, intracerebroventricular, intrathecal, and intraparenchymal routes[44] (Fig. 5A, B).

#### The BBB in Parkinson's disease

The pathophysiology of Parkinson's disease involves various potential pathways that can progressively worsen over time, leading to symptoms such as body tremors, muscle stiffness, unsteady gait, and difficulties with physical balance and coordination[45] (Fig. 5C, D). The primary pathological features of Parkinson's disease (PD) are the loss of dopaminergic neurons and the deposition of Lewy bodies. Lewy bodies are primarily composed of aggregated α-synuclein, which triggers the inflammatory response associated with Parkinson's disease. Monomeric α-synuclein stimulates the release of inflammatory mediators from brain pericytes, resulting in endothelial barrier dysfunction in rats[46]. One study found that fibrillar α-synuclein induced endothelial barrier dysfunction in the human brain when co-cultured with neurons[47]. Additionally, BBB leakage of fibronectin and hemosiderin was observed in the striatum of patients with Parkinson’s disease[48]. Minor blood–brain barrier disruption was also noted in the substantia nigra, white matter, and posterior cortical regions of patients with Parkinson’s disease[49]. The complexity of Parkinson's disease etiology necessitates a personalized approach to treatment. Ongoing research seeks to reduce side effects and create more targeted treatment strategies.

### The BBB in stroke

**Fig. 6:**
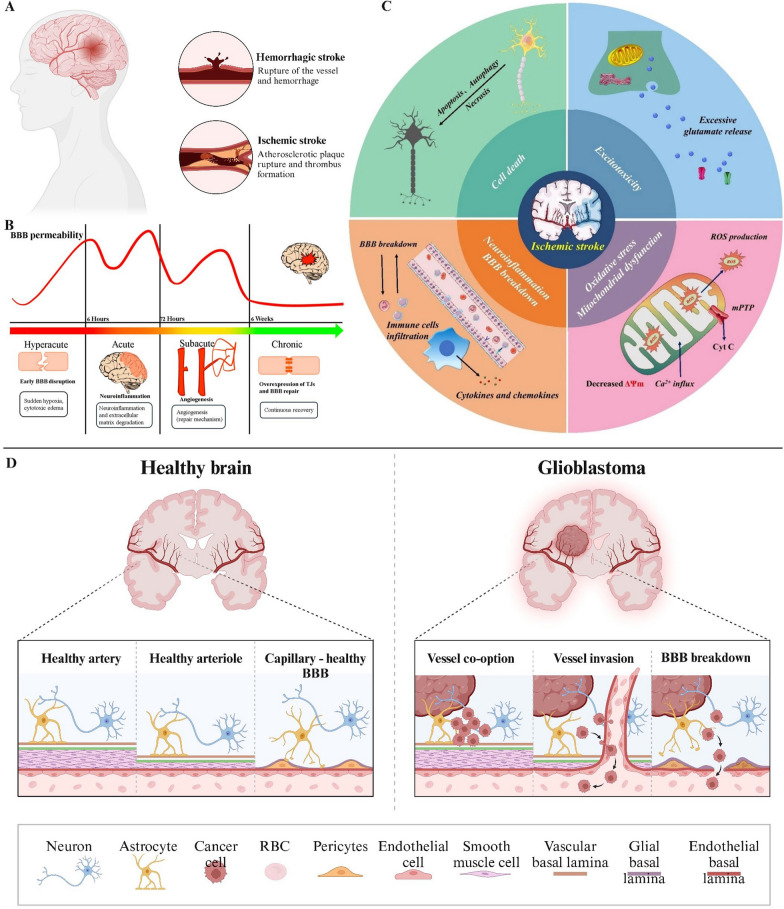
**A** Two common types of strokes are hemorrhagic stroke and ischemic stroke. Created in BioRender. **B** The pathogenesis of stroke is divided into hyperacute, acute, subacute, and chronic. **C** Various pathophysiologic changes associated with ischemic stroke, including glutamate excitotoxicity, neuroinflammation, BBB disruption, oxidative stress, mitochondrial dysfunction, and cell death. Reprinted permission from Ref.[54]. Copyright 2024, Springer Nature. **D** Microstructure of a healthy brain compared to a brain with glioblastoma. The blood–brain barrier is disrupted in brains with glioblastoma. Created in BioRender

Stroke is a highly disabling and often fatal brain condition (Fig. 6A, B, C). It is divided into two main types: hemorrhagic stroke and ischemic stroke, with ischemic stroke being more prevalent[50]. At present, effective clinical treatments for stroke are elusive, partly due to the challenges posed by the BBB, which hinders the delivery of medications to the damaged brain regions[51]. During the pre-stroke phase, sudden brain hypoxia damages the BBB, causing neuroinflammation, cytotoxic edema, production of reactive oxygen species, breakdown of tight junctions and the extracellular matrix, and increased BBB permeability. In the acute phase, the neuroinflammatory response worsens BBB injury by degrading tight junctions and the extracellular matrix, promoting neuroglial proliferation, and activating cardiomyocytes, thereby further increasing its permeability. In the subacute phase, notable repair mechanisms, including angiogenesis, start to take place. Brain repair and BBB permeability are strongly linked to lesion volume and stroke severity, as well as to neuroinflammation characterized by activated microglia and inflammatory cytokines[52]. Bernardo-Castro et al. discovered that the BBB exhibited its highest permeability 3–10 days after stroke. This phenomenon may be attributed to regenerative mechanisms, and this period of heightened permeability is linked with clinical recovery[53]. Various pathophysiologic changes are associated with ischemic stroke, including glutamate excitotoxicity, neuroinflammation, BBB disruption, oxidative stress, mitochondrial dysfunction, and cell death[54]. Understanding the various pathophysiologic changes associated with ischemic stroke contributes to the design and development of therapeutic diagnostic methods.

### The BBB in brain tumors

Brain tumors are among the deadliest and most aggressive CNS diseases globally. Its high rate of recurrence and the degree of difficulty in achieving a complete cure result in a reduced survival rate for patients. In individuals with brain tumors, the BBB undergoes significant structural and functional alterations compared to the intact barrier in healthy brain tissue[55]. The most aggressive brain tumor is glioblastoma multiforme (GBM), characterized by an exceptionally low cure rate. However, some nanodrug carriers with diameters of 1–100 nm have greater therapeutic and diagnostic significance for GBM[56] (Fig. 6D). In patients with this tumor, the BBB is primarily disrupted in the tumor core, where microvascular proliferation results in developing new, leaky blood vessels. In the peripheral regions of the tumor, the BBB maintains relative biological integrity[57]. As the primary tumor proliferates, neovascularization and intratumorally vascularization deteriorate, resulting in damage to the BBB. The BBB structure and function in GBM patients are markedly different from the normal BBB and are referred to as the blood–brain tumor barrier (BBTB). The BBTB is created by brain tumor capillaries located between tumor cells and is composed of specialized endothelial cells within the tumor vasculature[58]. Much like the BBB, the endothelial cells of the BBTB also express drug efflux transporter proteins[59]. The BBTB causes greater accumulation of waste metabolites and water in the neural parenchyma, which raises both intracranial and interstitial pressures. This forms a physical barrier that obstructs drugs from penetrating the brain parenchyma[60]. Although, in many cases, BBTB can severely limit drug transport, these alterations in BBTB enhance the penetration of chemotherapeutic agents compared to intact BBB[61]. In summary, understanding the distinctive characteristics of the BBTB could provide a crucial foundation for devising strategies to effectively bypass this barrier and improve drug delivery to brain tumors.

## Factors affecting nanoparticle permeability

**Fig. 7:**
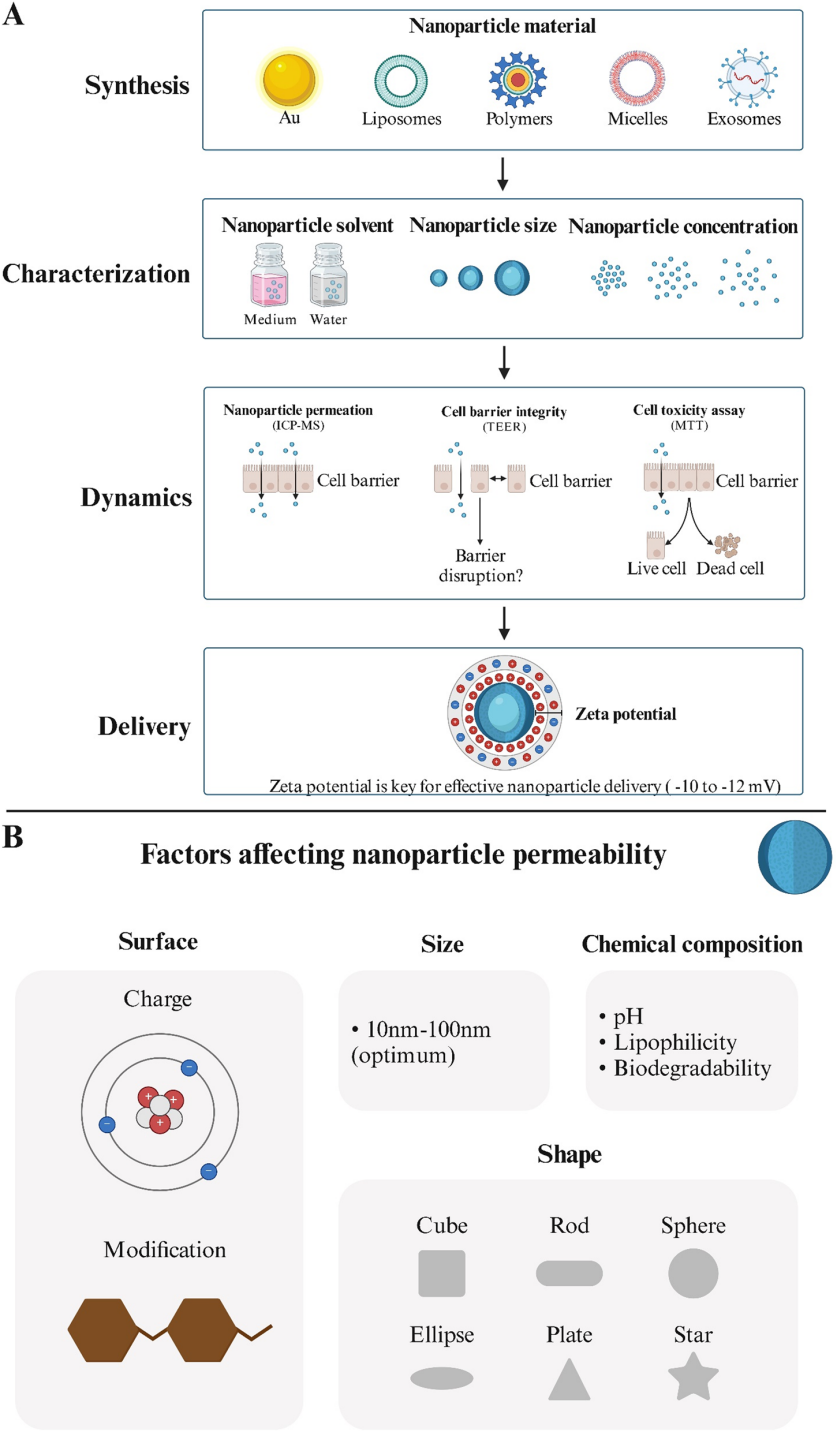
Physicochemical properties of nanocarriers. **A** Design development and evaluation processes for nanomedicine carriers. Created in BioRender. **B** Factors influencing the permeability of nanoparticles, including surface, size, shape, and chemical composition. Created in BioRender

The field of nanomedicine has seen rapid advancement in recent years, with increasing use of nanomaterials for disease treatment, notably as carriers for drug delivery[62]. The design of nanodrug carriers consists of four main stages. The physicochemical properties of nanocarriers mainly include size and shape of nanocarriers, chemical composition (lipophilicity, biodegradability, pH), surface charge, and surface modification (Fig. 7). These properties play a crucial role in determining their pharmacokinetic properties as well as in the range of biomedical applications.

### Size and shape

The size of nanoparticles is a critical factor influencing their ability to permeate the BBB. Studies have shown that the smaller their size, the more permeable they are to the BBB. However, nanoparticles smaller than 5 nm are more easily eliminated by liver filtration[63]. Nanoparticles larger than 200 nm are essentially unable to pass through the BBB[64]. Hence, nanoparticles ranging from 10 to 100 nm are commonly employed in studies aimed at drug delivery across the BBB. Not only does it improve permeability to the BBB, but it is also not eliminated by renal filtration[65]. Moreover, nanoparticles must navigate through the brain's extracellular space after traversing the BBB, which constitutes approximately 20% of the brain's total volume and is generally around 20 nm wide. So, larger nanoparticles would be limited by the extracellular space of the brain[66]. However, after testing the permeability of different sizes of carboxylated polystyrene nanoparticles to the BBB through an in vitro blood–brain barrier model based on μHuB, Nowak et al. found that 200 nm NPs were 10 times more permeable than 100 nm[67]. This proves that while smaller nanoparticles are more likely to cross the BBB in most cases, it is not absolute. The structure of different nanoparticles affects their size properties, so different nanomedicine carriers should be targeted in size design to make them perform better.

Similarly, the shape of the nanoparticles is very important for the penetration of the BBB. In most cases, nanoparticles are sphere but can also be built into other shapes, such as rod, cube, ellipse, and plate[68]. Rod-shaped nanoparticles adhere to the brain endothelium more readily than spherical nanoparticles, causing them to accumulate more in the brain[67]. The shape of the nanoparticles also affects their biodistribution, ability to be transported across endothelial cells, and the rate at which they are cleared[69]. Fu et al. systematically evaluated the effect of the physical aspect ratio of up-conversion nanocrystals on their cellular uptake properties and found that up-conversion nanocrystals with an aspect ratio of 2 exhibited the highest cellular internalization efficiency and were much less toxic to cells[70]. This suggests that NPs of different shapes are important for the design as well as the application of nanodrug carriers.

### Chemical composition

The BBB is made up of endothelial cells that are highly lipophilic. Thus, nanoparticles with lipophilic properties can cross the BBB more efficiently and are able to deliver drug molecules adequately into the brain parenchyma[71, 72]. Recently, researchers have created various drug delivery systems for the brain utilizing lipid nanoparticles. For example, solid lipid nanoparticles (SLNs), nanostructured lipid carriers (NLCs), liposomes, and noisome[73]. The biodegradability of nanoparticles is crucial in influencing their drug release rate and controlling pharmacokinetics. This reduces unnecessary intracerebral drug accumulation, prevents drug side effects, and allows the nanoparticles to have better biocompatibility[74]. The chemical composition of the nanoparticles also determines their pH, which influences the permeability of the BBB. At a plasma pH of 7.4, weakly basic drugs exist mainly in a non-dissociative form, and nanocarriers carry weakly basic drugs more easily across the blood–brain barrier. Therefore, the chemical composition of different nanoparticles will directly affect their permeability to BBB and whether they have higher biocompatibility and long-term efficacy.

### Surface charge

Another key factor in regulating the permeability of nanoparticles to the BBB is the surface charge. Surface charge affects the uptake of nanoparticles in the blood circulation by peripheral brain tissues and the interaction of nanoparticles with endothelial cells. It has been shown that cells have a higher uptake rate for positively charged nanoparticles than for negatively charged or neutral nanoparticles[75]. Neutral nanoparticles are about 100 times less permeable than positively charged nanoparticles[76]. Since endothelial cells contain more proteoglycans with negative charges, positively charged nanoparticles are more permeable across the BBB. However, neutrally charged nanoparticles diffuse faster in the brain and can rapidly deliver drugs to diseased sites[77]. Positively charged nanoparticles have the potential to generate reactive oxygen species, which can harm cells and trigger necrosis or apoptosis[78]. Macrophages also showed higher uptake and clearance of positively charged nanoparticles[79]. Therefore, by carefully adjusting the nanoparticle surface charge, it is possible to balance increased BBB permeability with reduced biotoxicity. Chen et al. synthesized a series of mesoporous silica nanoparticles (MSNs) with different charges by surface modification of MSNs. Experiments revealed that negatively charged MSNs could function as nanocarriers capable of crossing the BBB and delivering drugs without relying on external stimuli or ligand/receptor protein interactions[80]. Poly ethylenimine (PEI) and poly amidoamine (PAMAM), both positively charged polymers, enhance BBB permeability but are toxic to cells like red blood cells and neurons in the nervous system. In summary, modulating the surface charge of nanoparticles can improve their ability to deliver drugs into the CNS via the endogenous transport mechanism of the BBB.

### Surface modification

Surface modification or conjugation of active functional groups to nanoparticles is another strategy to modulate their permeability to the BBB when optimizing the nanoparticles' own physicochemical properties is not sufficient to achieve the desired effect. Nanoparticles have a large specific surface area and are well suited for functionalization with ligands or active functional groups to enhance their targeting ability[63, 81]. A drug-carrying nanoparticle based on pterostilbene (Pte) and black phosphorus (BP) was developed by Yin et al. Polydopamine (PDA) was applied to modify it, resulting in the formation of the BP-Pte@PDA delivery system. This delivery system specifically degrades and releases the drug in ischemic brain regions, significantly reducing infarction, improving neurological function, and inhibiting apoptosis[82]. Qi et al. created a dual-modified liposome using lactoferrin and musk, which effectively crossed the BBB and enhanced the brain-targeting efficacy of docetaxel, resulting in more potent glioma treatment[83]. Wiwatchaitawee et al. coupled polyethylene glycol (PEG) with PAMAM or PEI, which could mitigate their toxicity, and then evaluated biodistribution in a healthy mouse model, which was measured to be highly active and safe[84]. These examples show that functionalized nanoparticles with surface modification have better biocompatibility and drug-carrying capacity and can effectively treat various diseases.

## Types of nanocarriers

Nanoparticles are divided into two main categories: organic nanoparticles and inorganic nanoparticles. Organic nanoparticles, mainly polymer and lipid nanoparticles, hold great promise for delivering drugs into the brain. Inorganic nanoparticles mainly include metal-based nanoparticles and semiconductor nanoparticles. Metal-based nanoparticles are more controllable in terms of particle size, but they are not biodegradable and are often accompanied by toxicity.

### Organic polymer nanoparticles

Polymer nanoparticles are particles with a particle size between 10 and 1000 nm. These NPs are mainly synthesized from polymers with specific biocompatibility and biodegradability.

#### Poly (lactic-co-glycolic acid)

Poly (lactic-co-glycolic acid) (PLGA) is a copolymer made from the combination of polylactic acid and polyglycolic acid. It received FDA approval for its excellent biocompatibility, biodegradability, biosafety, and versatility and has become one of the most successful polymers in the biomedical field[85]. The ratio of lactone to hydroxyacetic acid can be adjusted to control the hydrophobicity and crystallinity of PLGA. The physicochemical properties of PLGA, including mechanical strength, solubility, hydration rate, and hydrolysis rate, are significantly affected by its degree of crystallinity and hydrophobicity[86]. PLGAs with greater crystallinity demonstrate enhanced mechanical strength, though they experience slower rates of hydration and hydrolysis. Studies have shown that specific concentrations of PLGA nanoparticles are not toxic to cells. Because the survival rate of both cell lines with added PLGA nanoparticles was more than 75%, this suggests that oral administration of PLGA nanoparticles does not adversely affect mice. Tissue distribution analysis in mice reveals that most PLGA nanoparticles are found in the brain, demonstrating their potential for drug delivery to the brain[87]. PLGA nanoparticles can cross the BBB either by passive diffusion or via active endocytosis. Unmodified PLGA nanoparticles predominantly crossed the BBB via passive endocytosis, influenced by particle size, but this route had limited permeability. Surface-modified PLGA nanoparticles can traverse the BBB through adsorptive-mediated transcytosis, carrier-mediated transcytosis, and receptor-mediated transcytosis. PLGA nanoparticles can extend their short half-life by combining with polyethylene glycol to create PLGA-PEG copolymer nanoparticles. The copolymers could also enhance their targeting ability across the BBB to release drugs to specific sites. Yusuf et al. developed PLGA thymoquinone nanoparticles coated with Polysorbate-80, which are designed to direct thymoquinone to brain lesion sites for treating Alzheimer's disease[88]. Meng et al. developed PLGA NPs loaded with Huperzine A and co-modifying it with lactoferrin enhanced its trans nasal delivery to the brain[89]. In conclusion, PLGA NPs are more biocompatible as well as tolerable than other NPs and can deliver drugs to the brain parenchyma through multiple pathways across the BBB depending on the functionalization mode. However, the lack of targeting of PLGA NPs and the wide variation in brain uptake efficiency by different routes of administration limit the therapeutic efficacy of loaded drugs.

#### Chitosan nanoparticles

Chitosan is a cation-carrying polymer with good biocompatibility and degradability. It is derived from the chitin of crustaceans and the fungi cell walls[90]. Chitosan nanoparticles are different from other biodegradable nanoparticles in that they can be biodegraded by a variety of enzymes contained in the human body, such as chitosanase and lysozyme[91]. All the products of biodegradation have been shown to be non-toxic, non-immunogenic, and non-carcinogenic, which is important for the biocompatibility of CNS drug delivery[92]. The attachment of chitosan to the surface of nanoparticles improves their biological and physicochemical properties. For example, chitosan can increase or restore the zeta potential of nanoparticles, which may confer a higher biological interaction of nanoparticles with anionic cell barriers. Chitosan also increases the hydrophilicity of the nanoparticles, which contributes to the enhancement of the NP stability in the aqueous environment. Chitosan can surface modify nanoparticles by creating new covalent bonds or chemical groups with the nanoparticles to improve their properties[93]. Saleem et al. created chitosan nanoparticles with a chrysin coating, known as Chr-Chi NPs. They discovered a protective mechanism by investigating how Chr-Chi NPs mitigate Aβ-induced neurodegenerative diseases. This protective mechanism is regulated by Chr-Chi NPs, which help preserve cognitive function and prevent neuronal death in the brain[94]. Haroon et al. prepared a nanocomposite by subjecting chitosan to a thiol substitution reaction and then linking centella asiatica to it. Under the ionic gelation method, the nanocomposite can cross the BBB via the nasal route and treat CNS diseases[95]. The limitation of chitosan nanoparticles is that their release rate is strongly affected by particle size and drug loading, and chitosan nano-delivery systems with higher drug loading can fail to pass through the BBB because of the large particle size.

#### Poly (ethylene glycol) and polyethylenimine

Poly (ethylene glycol) (PEG) has been widely used in the biomedical field due to it being highly biocompatible in physiological environments. Yin et al. improved the physiological stability and biocompatibility of gold nanoparticles by linking it to PEG[96]. By polymerizing other substances, the final polymer demonstrated excellent BBB permeability in a mouse model suffering from Alzheimer's disease. For polyethylenimine (PEI), Zhao et al. designed and synthesized a PEI-based drug delivery system that can be used for the targeted treatment of gliomas[97]. The polymeric system was able to cross the BBB and accumulate in the brain tumor region and provided a novel approach for imaging different types of cancers.

### Organic lipid nanoparticles

#### Solid lipid nanoparticles and nanostructured lipid carriers

Solid lipid nanoparticles represent a distinctive lipid-based nanocarrier system. It primarily consists of a solid hydrophobic lipid core and a drug that can be either hydrophilic or lipophilic[98]. SLNs can deliver drugs into the brain parenchyma through paracellular pathways, protein-mediated transport, adsorptive-mediated transcytosis, and receptor-mediated transcytosis. They play a crucial role in the process of the reticuloendothelial system. SLNs can enhance nasal drug absorption, bypass the BBB to deliver drugs to the CNS, and increase the concentration of active drug compounds in the brain[99]. Islamie et al. identified a bioactive component of centella asiatica extract, coumaric acid, which is toxicologically protective against neuronal cells. They made an SLN formulation of coumaric acid and administered it via the nasal route into the brain, which significantly improved the bioavailability and pharmacological activity of coumaric acid[100]. Curcumin SLN carriers were formulated using solvent evaporation by Campisi et al. They demonstrated that administration of curcumin-loaded SLNs in TgCRND8 mice inhibited the expression of TG2-S, thereby reducing the activation of the apoptotic pathway. It also increased levels of TG2-L, which played a restorative role in Alzheimer's disease models[101]. Saini et al. used chitosan-coated SLNs to release ferulic acid across the BBB, which could improve bioavailability by bypassing the hepatic first-pass effect and prolonging its presence in the body[102]. Nanostructured lipid carriers are a second-generation type of SLNs, composed of both solid and liquid lipids along with surfactants. It has a particle size between 50 and 300 nm and has the advantages of good biocompatibility, biodegradability, and high drug-loading capacity. Gartziandia et al. designed and developed an innovative chitosan-coated NLC formulation, suitable for safe brain drug delivery through the nasal route[103]. In conclusion, the drug delivery design of SLNs has controlled-release properties that allow for sustained drug delivery over a long period of time. This can improve drug stability and drug bioavailability, maintain drug concentration in plasma, and reduce drug toxicity. The limitations of SLNs are their low loading capacity for drugs and the possibility of drug efflux during storage, reducing efficacy. The limitations of SLNs are their low loading capacity of the drug and the possibility of drug efflux during storage, which reduces the efficacy. Nanostructured lipid carriers have poor stability, are prone to lipid crystallization, and have a complex formulation process that is difficult to apply widely.

#### Liposomes

Liposomes are synthetic or natural lipid bilayers that have a structure similar to biological membranes, which allows this type of nanoparticle to encapsulate hydrophobic and hydrophilic molecules separately or simultaneously[104]. Liposomes are highly biocompatible and biodegradable, making them suitable for drug delivery as well as immune responses[105]. Liposomes are categorized into multilamellar vesicles, small unilamellar vesicles, and large unilamellar vesicles. Ligands such as proteins, antibodies, and carbohydrates can be attached to the liposome surface through covalent or non-covalent bonds to facilitate targeting. Wang et al. constructed BV2 cell membrane-encapsulated polyethylene glycolized liposomes and delivered them to brain microglia via lymphatics, which ultimately inhibited Aβ-mediated neuroinflammation[106]. The liposome also provides sustained release of the drug, preventing oxidation and premature degradation. Passive targeting by liposomes can be achieved to minimize drug accumulation in healthy tissues, reduce toxicity, and improve local therapeutic effects[107]. Incorporating site-specific or tissue-specific ligands into the liposome can provide it with active targeting capabilities. This enables the liposomes to concentrate in specific tissues, thereby minimizing potential harm to non-target tissues. Additionally, liposomes can readily penetrate most biological barriers because their structure is similar to cell membranes, which enhances their BBB-crossing ability. However, liposomes still have limitations in that they have a low drug-carrying capacity and may lose their efficacy before they reach the site of disease. Synthetic liposomes are readily cleared by the reticuloendothelial system, and cholesterol-rich animal-derived liposomes impede receptor-mediated signaling and affect BBB permeability.

#### Micelles

Micelles are aggregates of surfactant phospholipid molecules dispersed in a liquid. Li et al. prepared borneol-modified schisandrin B micelles (Bor-Sch B-Ms) using a thin-film dispersion technique. The substance can accurately deliver the drug to the diseased area in the brain, effectively increasing the bioavailability of the drug. The study results demonstrated that modifications to the micelle surface enhanced drug uptake by bEnd.3 cells. This indicates that Bor-Sch B-Ms can enhance the therapeutic effects on N2a cells and facilitate a greater amount of drugs crossing the BBB into the brain parenchyma[108]. Lv et al. developed polymeric micelles conjugated with rabies virus glycoprotein 29 to co-deliver BACE1-shRNA and epigallocatechin-3-gallate. It improves intracerebral delivery by targeting specific receptors and neurotransmitters, and also shows strong antioxidant ability and effectiveness in alleviating neuroinflammatory responses[109]. However, the preparation process of micelles may lead to problems such as inhomogeneous particle size and poor stability. Under physiological conditions, the lack of stability of micelles can lead to dissociation and premature release of the loaded drug, which can reduce delivery efficiency and cause toxicity to the organism[110].

#### Exosomes

Exosomes (EXO) are endosomal-derived nano lipid vesicles between 30 and 150 nm in size. It is rich in nucleic acids, lipids, and proteins that mediate intercellular communication during normal physiological and pathological processes[111]. Hong et al. demonstrated that exosomal miR-233 mediated the crosstalk between Alzheimer's cell models and microglia. Anti-inflammatory microglia-derived exosomes deliver miR-233 to an Alzheimer's cell model, which in turn repairs neurological damage[112]. Wang et al. synthesized an olesoxime-resveratrol encapsulated in exosomes. The drug-loaded nanoparticles demonstrated excellent biocompatibility, successfully crossed the blood–brain barrier via intravenous injection without causing significant damage, and effectively inhibited Aβ1-42 aggregation, making them a promising treatment for Alzheimer's disease[113]. So, exosomes offer flexible surface properties, low toxicity and immunogenicity, great biocompatibility, and the capacity to reduce nucleic acid degradation. degradation. These properties make exosomes excellent carriers for delivering nucleic acids[114]. However, obtaining large quantities of exosomes is difficult because the extraction of exosomes requires a very complex process, so there is a need to develop methods to extract exosomes more easily.

### Inorganic nanoparticles

Inorganic nanoparticles, primarily consisting of metal and semiconductor nanoparticles, possess distinctive optical, electrical, and magnetic properties. Their biological properties can be enhanced by adjusting parameters such as size, shape, structure, and composition, while their surfaces can be functionalized with ligands and polymers[115].

#### Gold nanoparticles

Gold nanoparticles (AuNPs) are the most outstanding metal nanoparticles. It has antioxidant and anti-inflammatory properties that can improve damaged neurons. And it can also play a big role in restoring damage and treating neuronal inflammation, among other things. As a result, it can be utilized to treat various neurodegenerative diseases[116–118]. A distinguishing feature of AuNPs is surface plasmon resonance (SPR), an optical effect that arises when light strikes a metal surface[119]. For the most part, they do not undergo oxidative processes and play important roles in fields as diverse as pharmacology, sensing, and bioimaging. However, AuNPs are not degraded in the brain, and accumulating too many of them can lead to cellular mitochondrial damage and disrupt the integrity of the BBB[120].

#### Silver nanoparticles

Silver nanoparticles (AgNPs) have found extensive use in biomedical applications. It is also one of the most promising nanocarrier systems. This is because of their exceptional physicochemical properties, including a high surface area, straightforward synthesis and modification, and the ability to activate brain immune responses. AgNPs travel with the bloodstream to the BBB region mainly by binding to serum proteins in the bloodstream. Then it enters the brain parenchyma through transcellular pathways such as passive diffusion, carrier-mediated active transport, and endocytosis[121]. Some AgNPs with smaller particle sizes can cross the BBB via the paracellular pathway[122]. It has also been shown that AgNPs can enter the central nervous system directly via the olfactory or trigeminal nerves[123]. AgNPs ionize during internalization, leading to the release of silver ions that activate ROS production and cause mitochondrial damage, which can ultimately lead to cell death[124]. However, AgNPs produced through green and sustainable processes are less toxic and can be used to treat neurodegenerative diseases[125]. It also inhibits the production of foreign ROS without any substrate.

#### Selenium NPs

Green synthesized selenium NPs (SeNPs) can be used as nanocarriers for various disease treatments. Its strong bioactivity, biocompatibility, stability, and low toxicity have garnered significant interest from researchers in the field[126, 127]. SeNPs increase their ability to penetrate the BBB when bound to specific compounds. Functionalization of SeNPs with polyvinylpyrrolidone (PVP) and polysorbate 20 (Tween) by Kalčec et al. promoted the permeability of the loaded drug to the BBB[128]. This suggests that SeNPs possess the potential to cross the BBB for the treatment of CNS diseases when combined with specific compounds.

#### Mesoporous silica NPs and porous silicon NPs

In addition to their high biocompatibility, mesoporous silica NPs can be tailored to the size of particle size and surface pore size to determine the type of drug loading and to increase the drug loading capacity. Chen et al. introduced a ligand-free poly (ethylene glycol)-based variant of mesoporous silica NPs through tail surface modification with excellent BBB permeation[129]. It can inhibit brain tumor growth and attenuate drug-induced side effects. Pinna et al. made mesoporous silica NPs to resemble viral shapes and found that they could cross the BBB both in vivo and in vitro without any structural modifications to brain tissue[130]. This demonstrates that the use of mesoporous silica NPs for the treatment of CNS diseases does not cause serious side effects.

Porous silicon NPs are manufactured from silicon wafers by an electrochemical etching process. This process gives them a high surface area and enables them to be loaded with a variety of therapeutic agents such as nucleic acids, proteins, etc. Porous silicon NPs can be oxidatively hydrolyzed in aqueous solution under physiological conditions, and its degradation product, silicic acid, is non-toxic and excreted in the urine[131]. Shin et al. developed a drug-delivery system loaded with temozolomide based on porous silicon NPs to improve the survival of mice with glioma. temozolomide drug delivery system based on porous silicon NPs, which improved the survival rate of mice with glioma[132]. No accumulation of toxicity was detected in other healthy tissues in vivo, suggesting that porous silicon NPs have great potential in the field of drug delivery.

## Mechanism of nanoparticle penetration through the BBB

The blood–brain barrier often prevents drugs intended for treating CNS diseases from crossing the BBB, thereby hindering their accumulation and effectiveness in the brain. Recent advancements in nanomedicine have introduced innovative methods for delivering therapeutic agents across the BBB, utilizing both endogenous and exogenous transport mechanisms[31, 32], including various nanomedicine carriers.

### Endogenous transport mechanisms

#### Passive diffusion

**Fig. 8:**
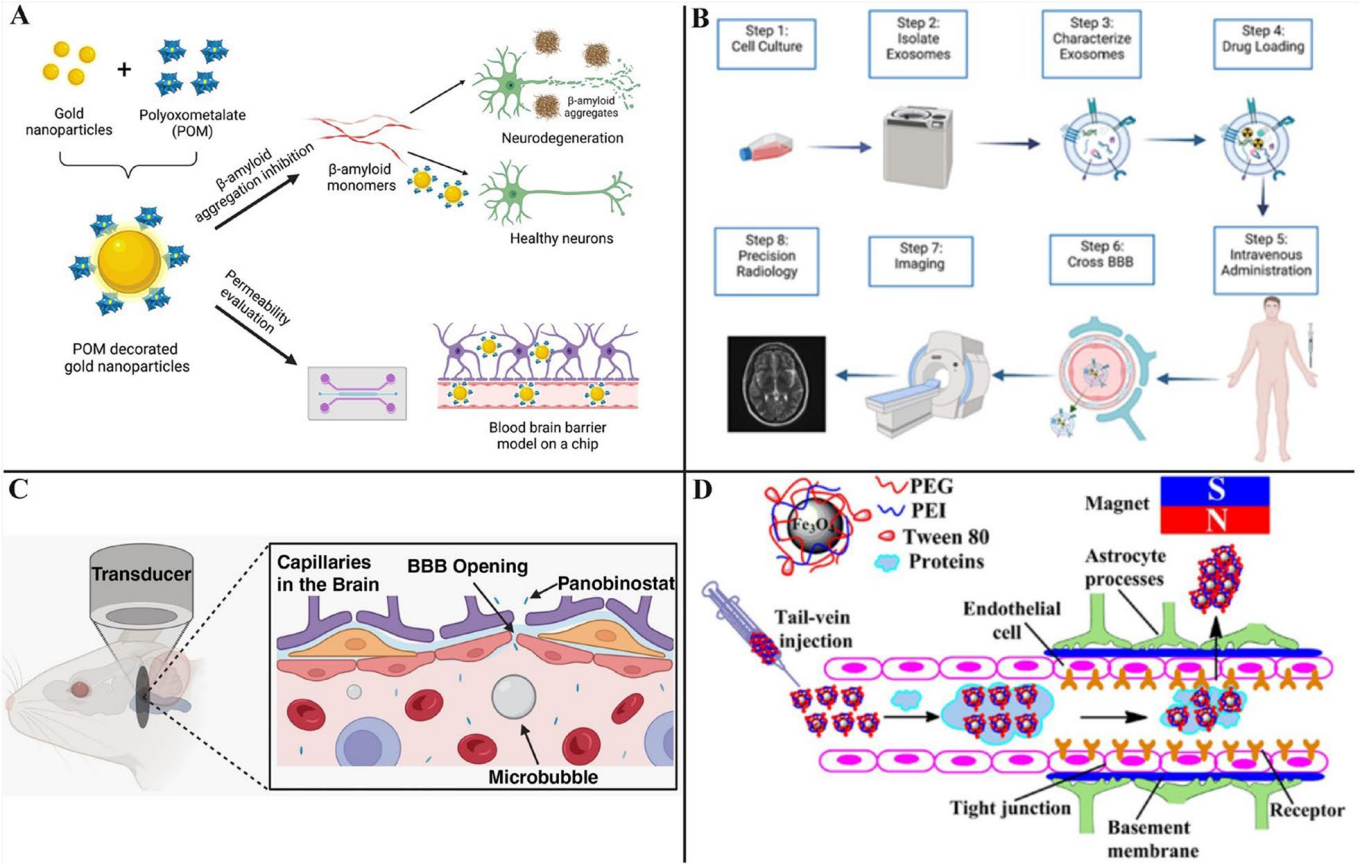
Examples of endogenous and exogenous transport mechanisms of nanoparticles across the blood–brain barrier. **A** The negatively charged three-component nanohybrid system AuNPs@POM@PEG inhibits Aβ aggregation and crosses the blood–brain barrier on the chip. Reprinted permission from Ref.[135]. Copyright 2023, Nanomaterials. **B** Exosomes can be used for precise imaging of the nervous system. Reprinted permission from Ref.[165]. Copyright 2022, The Authors. **C** Focused ultrasound and microbubbles can cause the BBB to open, allowing larger amounts of chemotherapeutic agents to enter the brain parenchyma. Reprinted permission from Ref.[176]. Copyright 2023, The Authors. **D** Superparamagnetic iron oxide nanoparticles coated with polyethylene glycol, polyethyleneimine, and polysorbate 80 can effectively cross the blood–brain barrier in the presence of an applied magnetic field. Reprinted permission from Ref.[181]. Copyright 2016, American Chemical Society

The tight junction’s gap between endothelial cells is 4–6 nm. This allows nanoparticles smaller than 4 nm, including gold nanoparticles and carbon dots (CDs), to pass through the BBB via passive diffusion[133, 134]. AuNPs are clusters of particles measuring between 1 and 100 nm, featuring gold cores enveloped in surface coatings. When suspended in a liquid, typically water, they are referred to as colloidal gold. Perxés Perich et al. designed an 18 nm negatively charged three-component nanohybrid system, AuNPs@POM@PEG, built on gold nanoparticles. The surface of this hybrid system is coated with polyoxometalates and polyethylene glycol, which enhances cellular internalization via passive diffusion and effectively inhibits Aβ aggregation[135] (Fig. 8A). Sokolova et al. investigated the changes in the rate and time of fluorescent ultrasmall gold nanoparticles (with diameters on the order of 3 nm) crossing the BBB by confocal laser scanning microscopy. They proposed that gold nanoparticles traverse the BBB via passive diffusion[136]. Zhou et al. demonstrated that their synthesized Y-CDs not only have hydrophilic surfaces, but also exhibit more hydrophobic functions through in vitro studies. The substance can also cross the BBB by passive diffusion[137]. In another study, Yan et al. combined Y-CD with the photosensitizer Ce6 to create the multifunctional nanocomponent yCD-Ce6. It was also shown that the multifunctional nanocomponent could cross the BBB after intravenous injection in the tail of mice[138]. In summary, these studies suggest that carbon dots represent a promising platform for nanodrug delivery. It can pass through the BBB by passive diffusion.

#### Carrier-mediated transcytosis

The blood–brain barrier has multiple transport protein systems. These transporter protein systems actively and selectively transport specific molecules, such as endogenous substances and nutrients. Examples include peptides, amino acids, and glucose, all essential for maintaining normal physiological function and metabolism in the brain. Glucose and amino acids cannot diffuse passively into the brain because of their polarity, so they are usually transported by carrier-mediated transcytosis. Examples include the glucose transporter, amino acid transporter, monocarboxylic acid transport system, and glutathione transporter[139–142]. The strategy for crossing the BBB using carrier-mediated transcytosis involves first determining whether the newly designed and synthesized molecule has a high affinity for the transporter protein. If it does, the molecule is then coupled to a drug carrier for experimental evaluation.

#### Adsorptive-mediated transcytosis

The first step in transcytosis is the endocytic uptake of nanoparticles. The process is divided into two main steps, where the nanoparticles are first adsorbed on the cell membrane and then internalized through energy-dependent pathways[143, 144]. This suggests that the strength of adsorption between the nanoparticles and the cell membrane determines the level of strength of adsorptive-mediated transcytosis. Based on this, researchers have designed and developed a series of drug delivery systems. y attaching cationic molecules (like chitosan and albumin) or using a cationic polymer core, drugs are encapsulated inside, and ligands are coated on the surface. This method enables the drug to cross the BBB and reach the brain parenchyma, where it can take effect[145–149].

In addition to cationic polymer cores, certain polysaccharides can also serve as positively charged materials. These polysaccharides can improve the adsorptive-mediated transcytosis process, thereby significantly enhancing drug permeability across the BBB. Dombu et al. identified that maltodextrin nanoparticles can attach to anionic sites on the cell membrane early in endocytosis and penetrate the BBB through a cholesterol-dependent exocytosis pathway[150]. Another polysaccharide, chitosan, exhibits excellent biocompatibility, strong adhesion, degradability, and low toxicity, among other beneficial properties. It can also enhance electrostatic interactions with cell surfaces and, importantly, possesses polycationic properties, making it a promising drug carrier for various applications[145].

#### Receptor-mediated transcytosis

Receptor-mediated transcytosis belongs to the most widely studied mechanisms of drug delivery via BBB transport nanocarriers[34]. The transport mechanism binds the drug first to the nanoparticles and then to the transporter protein, which transports the entire structure to the brain. During RMT, the substance is exposed to different environments. The pH of the different environments in which cells are located inside and outside of the cell can change significantly, so it is important that the nanomedicine carriers are able to maintain stability in these environments[151]. Compared to adsorptive-mediated transcytosis, specific binding sites are present in receptor-mediated transcytosis, and the affinity between ligand and receptor is higher. RMT begins with ligand-receptor binding, which is quickly followed by receptor-mediated endocytosis, delivering the drug carrier attached to the ligand to a specific location. In receptor-mediated drug delivery systems, the carrier's surface does not have to be positively charged. However, the delivery system's surface may have a neutral or negative charge, which helps to minimize cytotoxicity. Typically, to achieve receptor-mediated transcytosis, nanoparticles need to be conjugated to a specific ligand. It was shown that cRGD, H-ferritin, angiopoietin-2, apolipoprotein A1, and lactoferrin can all be conjugated to nanoparticles, thereby promoting their permeability to the BBB[152–157]. Certain ligand-modified nanoparticles have dual targeting capabilities, allowing them to both traverse the BBB and specifically target brain lesion sites. As the ligands on the nanocarriers bind to receptors on abnormal brain cells, these nanoparticles can act as a novel vehicle for more effectively crossing the BBB and treating CNS diseases.

#### Efflux pump

Efflux pumps are associated with the normal transfer and metabolism of substances in the cell, which can excrete the drug, reduce the concentration of the drug, and thus affect its efficacy. Brain endothelial cells feature efflux pumps on their surface, with P-gp being the most notable. Specific inhibitors of P-gp are employed to enhance the entry of nanocarriers into the brain and to minimize drug efflux[158]. Gomes et al. utilized transferrin-receptor peptide-functionalized nanoparticles, designed to target the BBB, as siRNA vectors to inhibit P-gp. After performing BBB cell-based modeling to assess permeability experiments on it, the nanoparticles were found to be able to increase the permeability of siRNA through the BBB twofold. It also successfully reduced the expression of P-gp mRNA by 52% after transfection[159]. The mechanism involves nanoparticles targeting the BBB to either induce or inhibit P-gp, resulting in reduced P-gp expression. This reduction increases the permeability of P-gp substrates at the brain membrane, allowing them to exert their therapeutic effects[160–162].

#### Intranasal drug delivery

Intranasal drug delivery is a method of delivering drugs directly to the brain, bypassing the BBB, which avoids the adverse effects that occur when drugs are administered to the brain by other routes and improves patient compliance. The nasal cavity consists of three main separate parts: the vestibular, respiratory and olfactory regions. The vestibular region contains a large amount of mucus and ciliated cells, which act as a barrier against foreign particles. The respiratory region consists of basal cells, goblet cells, and ciliated and non-ciliated columnar epithelial cells, and is the largest region of the nasal cavity[163]. The olfactory region is covered by olfactory cells and receptors. The olfactory receptors on the cilia in this region contact the nanoparticles and transmit different signals to the cribriform plate, forming connections with the olfactory bulb neurons[164]. Secondary neurons receive signals from the olfactory nerve, analyze them, and transmit them to the brain. The trigeminal and olfactory nerves are important for intranasal drug delivery to the brain, with trigeminal neurons transporting drugs from the nasal cavity to the pontine regions of the brain, with minimal effect on the olfactory and frontal lobes. The vascular structure of the respiratory region helps the nanoparticles to enter the bloodstream and thus indirectly the central nervous system[165] (Fig. 8B). However, the intranasal route of drug delivery still has some unresolved issues, such as the need to overcome cilia and mucosal clearance in the vestibular region, as well as enzymatic degradation after the introduction of nanotherapeutic formulations into the nasal cavity[166].

#### Natural cell membranes

In recent years, biomimetic nanoparticles based on natural cell membranes have been widely developed. Biomimetic nanoparticles are biocompatible and low-immunogenic drug delivery vehicles formed by encapsulating cell membranes from cells such as red blood cells and cancer cells on the surface of different nanoparticles. It can evade the immune clearance process of the body and prolong the circulation time of the drug in the body. And the nanoparticles encapsulated by different cell membranes can inherit the functions of the cell membrane source cells. Such as the homologous targeting effect on cancer cells, which could make this drug delivery system more effective in targeting cancer treatment[167]. Gao et al. wrapped PLGA nanoparticles loaded with curcumin in red blood cell membranes and found that nanoparticles encapsulated in red blood cells could prolong the blood circulation cycle and avoid the immune clearance process through targeting experiments in mice with Alzheimer's disease[168]. This demonstrates that cell membrane-based biomimetic nanoparticles have a very promising future in the treatment of CNS diseases.

### Exogenous transport mechanisms

#### Focused ultrasound

Focused ultrasound (FUS) is a non-invasive technique that temporarily disrupts the BBB. This technology shows considerable potential for improving intracerebral drug delivery and treating central nervous system diseases[169]. The technology utilizes microbubbles that expand and contract when activated by low-intensity ultrasound energy. This repeated oscillation applies mechanical forces (sound pressure) to the endothelial cells of the BBB, loosening the tight junctions and briefly opening the barrier. Activation of microbubbles by FUS can be confined to specific brain regions, is reversible, does not damage brain tissue, and is, therefore, a controlled process[170]. FUS is currently FDA-approved for treating central nervous system diseases, including essential tremor and tremor-related Parkinson's disease. And it is being tested for the treatment of epilepsy, neuropathic pain, and psychiatric diseases[171–173]. Magnetic resonance imaging (MRI)-guided FUS can precisely target nanomedicine carriers to specific sites in the brain without the need for craniotomy[174]. Transcranial FUS is often combined with intravenous microbubbles (MBs), which can reduce the negative effects of frequent FUS on brain tissue[175]. This combined approach permits larger amounts of drug to enter the brain parenchyma to accumulate and exert efficacy[176] (Fig. 8C). MBs oscillate steadily in a specific region, generating shear and circumferential stresses on the microvascular wall of the BBB, which can open the BBB transiently. However, the BBB will regain its integrity after 4–6 h[177]. During the blood–brain barrier opening, nanocarriers loaded with therapeutic drugs can penetrate the BBB and deliver the drugs to the brain parenchyma. MRI results showed that the BBB, which was opened by FUS, closed naturally without symptoms such as bleeding or infarction. This suggests that a temporary BBB open drug delivery strategy can be effectively and safely performed using this method[178]. At high sound pressures, FUS induces large volume oscillations in microbubbles, a phenomenon known as “inertial cavitation”. Inertial cavitation leads to the eventual collapse of the microbubbles, creating jets, shock waves, or other inertial effects that have the potential to damage the brain parenchyma and the blood–brain barrier[179].

#### Magnetic field force

Magnetic nanoparticles (MNPs) feature a magnetic iron oxide core and are encased in biocompatible coatings like dextran, lipids, or polymers. It has been shown that external magnetic fields can be used to migrate MNPs from the vascular lumen into the brain parenchyma. Magnetism can activate the paracellular transport pathway by temporarily disrupting intercellular connections through internalized MNPs[180]. Huang et al. prepared superparamagnetic iron oxide nanoparticles coated with polyethylene glycol, polyethyleneimine, and polysorbate 80 (Tween 80). Placement of the polymer under an external magnetic field via the caudal IV route effectively crosses the intact BBB[181] (Fig. 8D). Chen et al. investigated the question of whether exposing MNPs to external static electromagnetic fields could increase their permeability to the BBB. They found that an external static electromagnetic field increased the penetration of MNPs into the BBB to 8.47%, which was 5.11% higher than the penetration without being under a static electromagnetic field[182]. Magnetic nanoparticles also serve as MRI contrast agents and drug carriers in Alzheimer's disease. An increasing body of research indicates that the severity of Alzheimer's disease is more closely associated with pathological features of tau than with Aβ plaques. Hou et al. found that D-TLKIVWC (7-DP) was a D-cysteine extension of 6-DP. It both inhibits tau aggregation and protects nerve cells from tau-induced toxicity. They attached 7-DP to magnetic nanoparticles to enable the drug to cross the BBB and target the CNS more effectively[183]. Therefore, drug-loaded MNPs are promising for the treatment of CNS diseases when crossing the BBB through the action of an external magnetic field. However, the accumulation of excess MNPs in the brain may also cause toxic buildup and, thus, adverse effects.

#### Laser

Research on laser therapy has led to the development of three methods of CNS-targeted drug delivery using lasers to increase BBB permeability. These are laser interstitial therapy (LITT), photodynamic therapy (PDT), and photo biomodulation therapy (PBM). LITT can cause localized damage to the BBB by converting light energy into thermal energy through a probe. Leuthardt et al. evaluated 14 patients treated with LITT and found that BBB permeability to drugs peaked 1–2 weeks after LITT treatment and recovered after 4–6 weeks[184]. However, the heat generated by the therapy is difficult to control, which may trigger high-temperature damage to nearby organs or tissues. To overcome this difficulty, magnetic resonance technology can be used to guide LITT, thus avoiding thermal damage[185].

The mechanism of action of PDT is the same as that of PBM, which is to produce cytotoxic reactive oxygen species by activating the photosensitizer with specific wavelengths of light, and then destroying the tight junction proteins to increase the permeability of the BBB. The difference is that PDT requires an exogenous photosensitizer, which produces higher toxicity and causes cellular damage, while PBM uses an endogenous photosensitizer, which is less toxic to cells[186]. However, the limitations and efficacy of laser therapy need to be further addressed before it can be used in clinical treatment.

#### Cell-mediated transcytosis

**Fig. 9:**
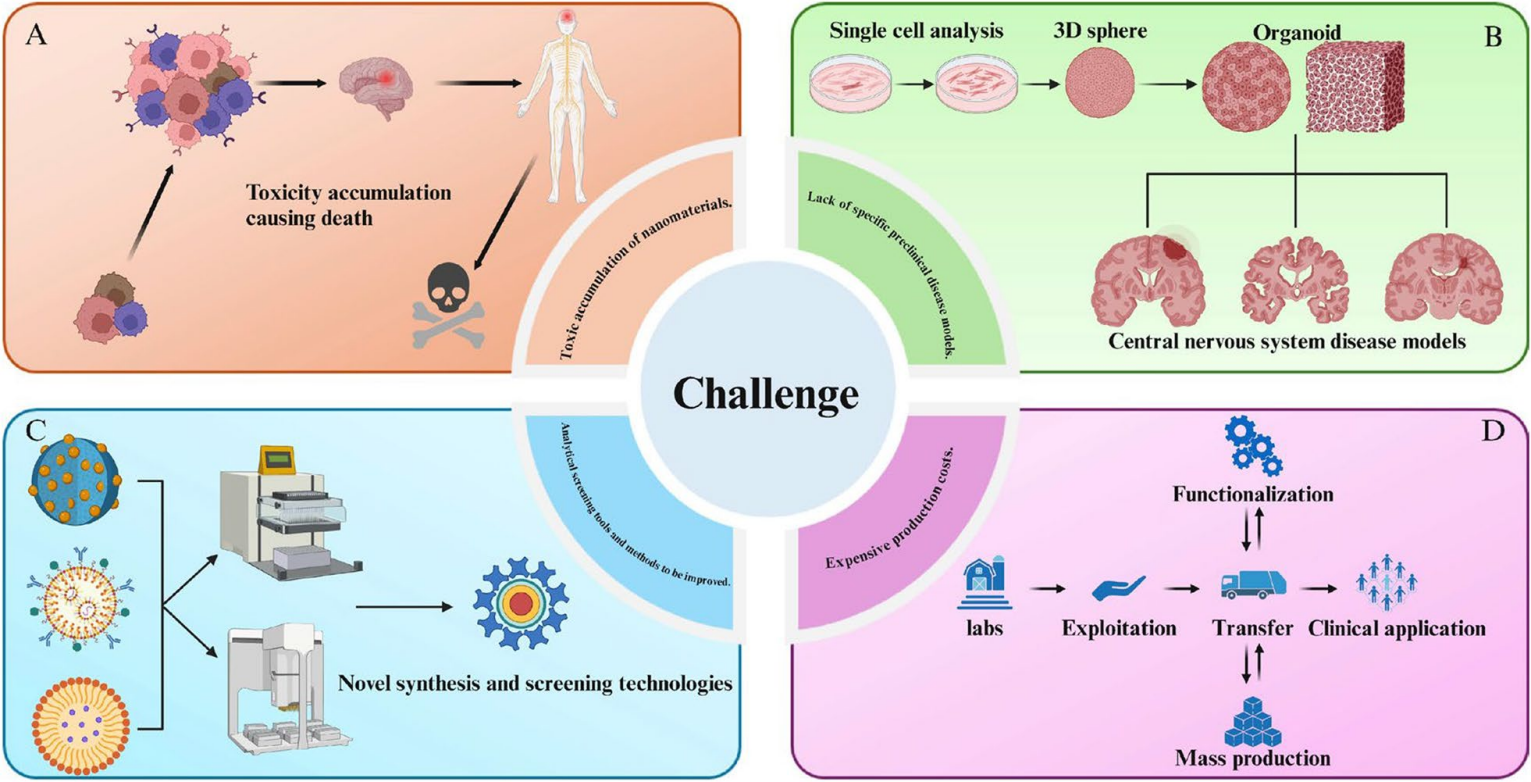
Existing challenges. **A** Toxic accumulation of nanomaterials. Created in BioRender. **B** Lack of specific preclinical disease models. Created in BioRender. **C**Analytical screening tools and methods to be improved. Created in BioRender. **D** Expensive production costs. Created in BioRender

Cell-mediated transcytosis is a common mechanism for immune cells to traverse biological barriers. In the case of neuroinflammatory and neurodegenerative diseases involving neuroinflammation, immune cells like macrophages and monocytes are well-suited for transporting treatments to the brain. This is due to their recruitment across the BBB during brain inflammation episodes. During strong autoimmune responses or central nervous system infections, immune cells like leukocytes, monocytes, and macrophages can cross the BBB using paracellular and transcellular routes[187]. In a recent study, Zhao et al. created a transgenic model to investigate Parkinson's disease. In the early phase of this disease model, macrophages that produce neuroglia-derived neurotrophic factors can offer protective support to the CNS. The fact that these macrophages can restore motility even in the late stages of the disease model indicates that immune cells could be effective drug carriers across the BBB[188] (Fig. 9).

## Challenge

Different types of nanoparticles have been employed as drug carriers to cross the BBB for treating CNS disease, resulting in notable progress. However, there are still several outstanding issues in this area that have not been adequately addressed.

### Toxic accumulation of nanomaterials

As nanomedicine carriers deliver drugs to the brain parenchyma, more nanoparticles accumulate in the brain or produce toxic metabolites, which may have side effects on specific organs and cause health problems in the organism. For example, some inorganic nanomaterials (gold nanoparticles, iron nanoparticles, and silica nanoparticles) are not easily metabolized in the brain. They worsen neurodegenerative diseases by causing damage to mitochondria, leading to redox imbalances, fragmenting the cytoskeleton, and generating inflammatory responses[189, 190]. Prolonged accumulation of silver nanoparticles in the brain produces oxidative stress, autophagy impairment, and inflammatory response resulting in neurotoxicity[191]. Some biodegradable functionalized nanoparticles can also be neurotoxic. For example, Yuan et al. introduced polysorbate 80-modified chitosan nanoparticles into the rat brain, and after a period of time, found that the rat brain cells died[192]. Secondly, although most nano-formulations are injectable, nano-formulations for neurodegenerative diseases are mainly in oral dosage form, so their acute as well as long-term neurotoxicity must be further optimized. In addition, the number of nanoparticles used in nano pharmaceutical formulations with their loaded drug concentration can also have a great impact on the accumulation of toxicity. Adjusting the appropriate ratio will greatly aid in the design and development of nano pharmaceutical formulations for safety. Nano pharmaceutical carriers must be biocompatible and able to degrade rapidly and efficiently in vivo into non-toxic and easily eliminated metabolites.

### Lack of specific preclinical disease models

The in vitro BBB models, as well as disease models that exist today, are poorly predictive tools. The expression of receptors and transporter proteins is heavily influenced by the culture conditions, which can lead to varying outcomes. Animal disease models are also flawed because of the inherent differences in the physiological nature of the BBB between animals and humans, so there is no way to tell if the results of experiments performed in animals are applicable to humans[193]. In addition, the characteristics and interactions of human CNS diseases are too complex to construct complete mechanistic models in vitro, but only by constructing animal models of such diseases[194]. Thus, even if successful in animal disease models, the drug delivery and therapeutic efficacy of this nanocarrier in the human body may be low, thus limiting clinical translation. For example, Loureiro et al. found that monoclonal antibodies derived from mice are cleared by the human immune system, which limits the efficacy of nanocarriers conjugated to this antibody[195]. Thus, utilizing animal disease models to optimize and design nanomedicine carriers remains challenging. Advancing the development of in vitro disease and blood–brain barrier models is crucial for overcoming these difficulties.

### Expensive production costs and the need to optimize analytical tools and methods

The technology and instrumentation required to manufacture nanomedicine carriers is very expensive. Operations such as surface modification and optimization of the produced nanomedicine carriers and testing their effects, can also be labor-intensive and costly. This has led to the need to develop new low-cost and more effective nanodrug carriers and further refine their manufacturing processes to enable large-scale production of nanodrug carriers. Cao et al. improved the flow stability of the integrated piston pump and the consistency of the nanoparticle micro-mixing process by integrating a pulsation damper into the microfluidic channel[196]. Thereby, a large number of PLGA nanoparticles loaded with antitumor drugs possessing consistent sizes were successfully produced, which promotes the stability and production volume of nanodrug carriers and reduces the cost of production wastage.

Most of the tools currently used to study the permeability of nanocarriers to the BBB, drug quantification, and their safety and efficacy assays are indirect or destructive. Examples include histological techniques, chromatographic techniques, etc., which can result in the inability to measure the specific dose of the active drug in the target site where the drug exerts its effect[193]. Although techniques such as micro-dialysis and cerebral open flow micro-perfusion allow for real-time, continuous, and accurate measurement of drug concentration and distribution of nanocarriers in the brain, they are highly dangerous to the human body. We need to standardize and make the characterization and analytical techniques safe to better evaluate the merits of nanocarriers used in clinical trials for drug delivery.

## Prospects

**Fig. 10:**
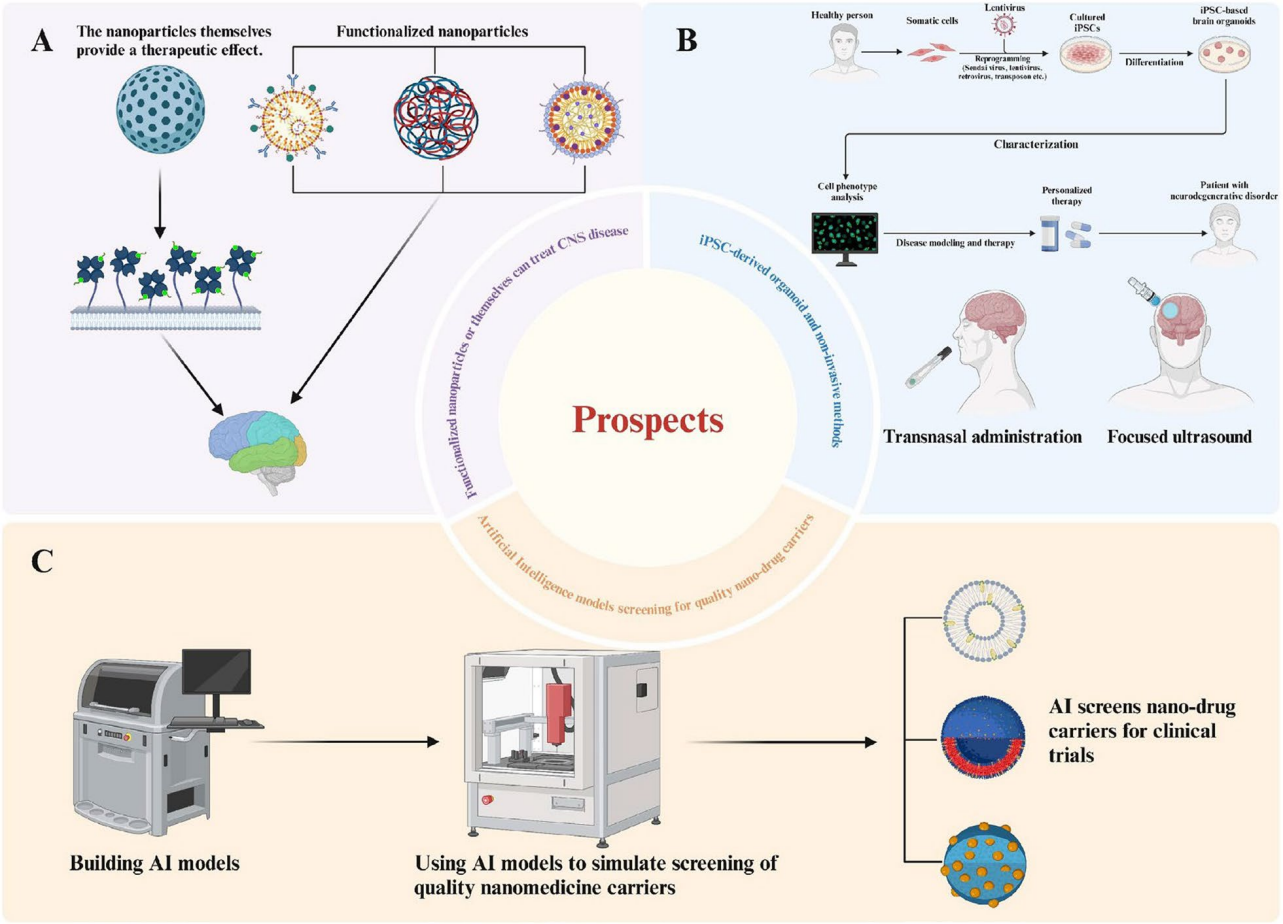
Further prospects for the field of nanoparticle-related drug carriers. **A** The nanoparticles themselves or functionalized nanoparticles have therapeutic effects on CNS diseases. Created in BioRender. **B** The iPSC-derived organoids that researchers are using to model and treat neurodegenerative diseases mimic the microenvironment of a patient's brain relatively perfectly for targeted therapy. Novel non-invasive methods include trans-nasal drug delivery and focused ultrasound techniques. Created in BioRender. **C** Artificial intelligence modeling to screen for high-quality nanomedicine carriers. Created in BioRender

The use of nanoparticles and their potential for therapeutic and diagnostic applications in CNS diseases has seen significant growth. A comprehensive understanding of the physiological properties of the BBB and how it alters under pathological conditions has also been investigated. Potential research directions could focus on the following areas (Fig. 10).

### Nanomaterials themselves as therapeutic drugs and more advanced nanomaterial design

While nanoparticles have been widely studied primarily as carriers for delivering drugs, the value of nanoparticles extends beyond designing them into drug delivery systems. This is because some NPs, including certain metal nanoparticles and PLGA nanoparticles, exhibit therapeutic properties independent of drug or coupling chemistry. They have been used in the past for treating neurodegenerative diseases, but their permeability to the BBB still needs to be further optimized[9, 197]. Designing a nanoparticle requires first determining its optimal physical and chemical properties, such as size, surface charge, and surface modifications. Further development of functionalized nanomaterials with enhanced stability, biocompatibility, and BBB permeability. Examples include carbon-based nanoparticles, liposomes, or polymer nanoparticles[198–201].

To address biocompatibility and safety issues, such as dealing with the toxicity of inorganic nanomaterials as they accumulate in the brain, these nanoparticles can be synthesized using more efficient and environmentally friendly processes. For instance, conjugating gold nanoparticles with PEG can greatly enhance the stability and biocompatibility of AuNPs[202]. And the drug carrier exhibits higher drug loading capacity, minimizing the systemic spread of toxicity during circulation by binding to pH-sensitive drug release. Integrating functionalized nanoparticles with additional techniques, like drug delivery systems and imaging modalities, can improve their effectiveness and extend their range of multifunctional applications. Encapsulating nanoparticles with specific ligands or loading therapeutic agents into nanocarriers can improve their capacity to deliver drugs selectively across the BBB. Employing functionalized nanoparticles as contrast agents can improve the resolution and accuracy of brain magnetic resonance imaging[203–205]. Focusing the research and development on more advanced nanoparticle optimization or design could further enhance the therapeutic diagnosis of CNS diseases. For biodegradable nanoparticles such as polysorbate 80-modified chitosan. we can appropriately elevate the percentage of chitosan in the system, which will further optimize its biodegradability and minimize neurotoxicity.

### An in vitro model of the blood–brain barrier for screening nanomedicine carriers

Several cell-based in vitro blood–brain barrier models have been developed in the past to predict the permeability of nano pharmaceutical formulations to the BBB[206, 207]. However, these 2D models fail to fully reproduce the unraveling complexity of the BBB, and there is still a need for accurate and physiologically relevant BBB models to facilitate the translation of nanomedicines for the treatment of CNS diseases from the laboratory to clinical applications. To better generalize the structure and function of the BBB, more complex in vitro 3D models can be developed. Static 3D models of the blood–brain barrier based on hydrogels, spheroids and organoids have been developed in recent years, and microfluidic-based chip models have also been attempted. However, to accurately assess the screening ability of these in vitro 3D blood–brain barrier models for nanomedicines, it is first necessary to validate their barrier function. Many drugs enter the BBB primarily through the transcellular pathway, so we can determine the physiological functional integrity of the in vitro model by immunofluorescence techniques to detect the amount of several membrane inward and outward transport proteins, such as GLUT-1, the transferrin receptor or P-glycoprotein[208].

We can also perform nanomedicine screening by constructing an in vitro blood–brain barrier model with CNS diseases. These disease models can incorporate the physiological and functional properties of healthy BBBs as well as the heterogeneity of different CNS diseases to enable personalized medicine. For example, the dynamic microfluidic device for brain tumors fabricated by Tricinci et al. using two-photon lithography can be successfully used as an in vitro model for high-throughput nanodrug screening[209]. Shin et al. developed a physiologically relevant microfluidic model of three-dimensional human neuronal cell culture that recapitulates the same key BBB dysfunction as in AD patients[210]. Thus, it provides a good platform for screening nano-novel drugs across the BBB for Alzheimer's disease. For a specific individual, it is also possible to simulate the microenvironment of that patient's brain relatively perfectly with iPSC-derived organoids for neurodegenerative disease modeling and therapy. With the continuous improvement of the above methods, nanomedicine carriers will solve the difficulties of all kinds of brain diseases and contribute greatly to the development of the biomedical field.

### Artificial intelligence-based drug delivery system for nanocarriers

With the rising global prevalence of neurodegenerative diseases, nanocarriers are garnering growing interest for their role in delivering drugs to the CNS. However, the screening of effective nanodrug delivery systems is a greater difficulty. This is because the number of combinations of nanoparticles and neurodegenerative disease drugs is enormous and involves a variety of assays. However, AI algorithms can speed up the process by screening the most promising candidate compounds for neurodegenerative disease drugs and NPs. González-Díaz et al. developed an AI detection model that could be used to detect effective nanodrug carrier systems. Recent studies have demonstrated that this AI assay model was a powerful tool in molecular science and neurodegenerative disease drug research for analyzing large datasets, including structural and non-structural parameters[211]. Examples of applications include drug screening, protein targeting tests, and functional prediction of drug release systems for encapsulated NPs[212–214]. Henser-Brownhill et al. developed computational models capable of predicting the clinically relevant physicochemical properties of nanocarriers and their mRNA payload delivery efficiency in human cells[215]. Deploying this computational model in large theoretical nanocarrier libraries enables rapid pre-screening of high-quality, high-performance nanocarrier candidates. This is then synthesized and validated by cell-based assays, which greatly reduces the time cost of preclinical development of nanomedicine carriers. Therefore, the use of artificial intelligence to assist in developing nano drug-carrying systems for treating central nervous system diseases has a broad development prospect.

## Conclusion

Research on nanoparticles and their application in CNS diseases has seen substantial growth, with increasing focus on utilizing nanomedicine carriers to efficiently deliver drugs across the BBB for treating these conditions. This review begins by discussing the discovery of the BBB and its physiological functions, and then provides a detailed description and illustration of its microstructure. The review then summarizes both endogenous and exogenous transport mechanisms for nanoparticles crossing the BBB. A thorough understanding of these theoretical foundations can aid in the development and design of nanomedicine carriers, ultimately enhancing their performance and therapeutic efficacy for CNS diseases. It also examines the factors influencing nanoparticle properties, such as size, shape, and surface charge. The review then outlines the different nanoparticle types, both inorganic and organic, and explores recent scientific advancements related to each category. It also explores the practical and potential applications of nanoparticles for crossing the BBB. Finally, existing challenges to brain drug delivery using nanoparticles are presented, such as the accumulation of nanoparticles in the brain, which can produce neurotoxicity, and the lack of preclinical CNS disease models that can mimic the microenvironment of the human brain relatively perfectly. Moreover, nanomedicine carriers are difficult to mass produce and use clinically due to the expensive cost of production as well as the complexity of the production process. Solutions are further proposed for these unresolved issues and provide prospects for the future development of optimizing and screening nanomedicine carriers for treating CNS diseases. This review seeks to synthesize previous experiences and examples to guide developing new diagnostic and therapeutic strategies for CNS diseases, as well as to enhance drug design for more effective delivery across the BBB. In conclusion, nanoparticles as brain drug carriers are a promising strategy for therapeutic diagnostics. It provides novel ways to optimize brain drug delivery strategies with high precision. Enhancing drug delivery to the brain by surmounting the BBB using this material is essential for advancing the diagnosis and treatment of CNS diseases. It is also a major challenge in modern nanomedicine.

## Data Availability

No datasets were generated or analysed during the current study.
